# Estimating Absolute Protein–Protein Binding Free Energies by a Super Learner Model

**DOI:** 10.1021/acs.jcim.4c01641

**Published:** 2025-02-20

**Authors:** Elton J. F. Chaves, João Sartori, Whendel M. Santos, Carlos H. B. Cruz, Emmanuel N. Mhrous, Manassés F. Nacimento-Filho, Matheus V. F. Ferraz, Roberto D. Lins

**Affiliations:** † Aggeu Magalhães Institute, Oswaldo Cruz Foundation, Recife 50670-465, Brazil; ‡ Laboratory for Applied Genomics and Bio-Innovations, Oswaldo Cruz Foundation, Rio de Janeiro 21040-900, Brazil; § Department of Fundamental Chemistry, Federal University of Pernambuco, Recife 50670-901, Brazil; ∥ Institute of Structural and Molecular Biology, University College London, London WC1E 6BT, U.K.; ⊥ Department of Computer Science, Princeton University, Princeton, New Jersey 08544, United States; # NEC OncoImmunity AS, Oslo 0349, Norway

## Abstract

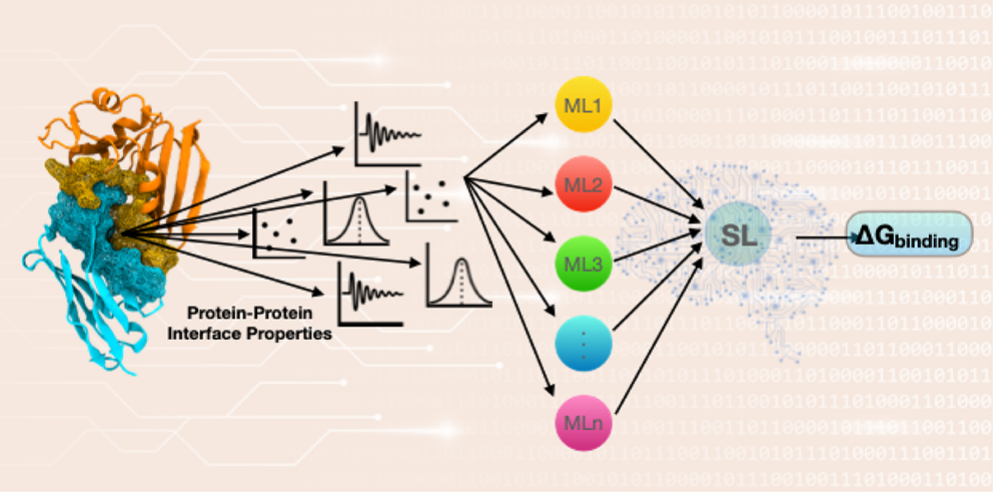

Protein–protein binding is central to most biochemical processes of all living beings. Its importance underlies mechanisms ranging from cell interactions to metabolic control, but also to *ex vivo* biotechnology, such as the development of therapeutic monoclonal antibodies, the engineering of enzymes for industrial biocatalysis, the development of biosensors for disease detection, and the assembly of artificial protein complexes for drug screening. Therefore, predicting the strength of their association allows for understanding the molecular mechanisms and ultimately controlling them. We devised a machine learning ensemble model that uses Rosetta-based quantities to predict binding free energies of protein–protein complexes with accuracy rivaling both computationally demanding methods and currently available ML/DL tools. The method was encoded into an application Python pipeline named PBEE, which stands for Protein Binding Energy Estimator, allowing a rapid calculation of the absolute binding free energies of protein complexes from their PDB coordinates.

## Introduction

Protein–protein association is central to most biochemical processes of all living beings. Its importance underlies mechanisms ranging from cell interactions to metabolic control but also to *ex vivo* biotechnology (e.g., biosensors and diagnostic kits). Understanding the underlying mechanisms and quantifying the strength of these interactions is crucial for advancing our knowledge of protein function, drug discovery, and the development of targeted therapeutic interventions through protein engineering (e.g., diagnostic markers^1^ and biopharmaceuticals^2,3^). Binding affinity can be satisfactorily assessed by the Gibbs free energy (Δ*G*^0^), a quantity that is experimentally challenging and resource-intensive to obtain. There are several molecular mechanics-based methods to accurately compute the absolute binding free energy () of proteins and small ligands.^4−7^ However, obtaining the  for protein–protein association has proven to be a harder task, likely due to the higher number of degrees of freedom, non-negligible entropic effects, and the involved time scale associated with binding events, which far exceeds what is achievable by conventional molecular simulations. Among the many methods published in the literature, enhanced sampling methods, such as metadynamics, are worth mentioning by their accuracy when computing protein–protein binding affinities. These calculations are highly computationally demanding, but Wang and collaborators have recently proposed a metadynamics protocol that was tested on 19 protein–protein structure complexes. The authors reported a standard error of 1.61 kcal/mol and a Pearson’s correlation of 0.84 from the experimental values.^8^ Nevertheless, the computational need associated with the technique precludes its use in a high-throughput fashion.

To overcome this issue, the community has increasingly turned to machine and deep learning algorithms in the past few years. Guo and Yamaguchi^9^ have reviewed the field and reported 6 structure-based models^10−16^ to predict protein–protein binding affinities. Nevertheless, most models have been developed to predict binding free energy of single and multiple point mutations considering small subsets of protein complexes using experimental data available in the SKEMPI2 database.^10−16^ As a consequence, these models are highly specific, and their performance varies significantly upon the test set used.

A more general approach was used by Vangone and Bonvin.^13^ They developed an ML model based on interface contacts and noninteracting surface to predict the binding energy of protein–protein complexes. Their approach is based on the evidence that a greater number of interfacial contacts correlates with stronger binding. The authors used a curated data set comprising 81 protein complexes from affinity benchmarks,^17^ reaching a validation accuracy of 1.89 kcal/mol RMSE and a Pearson correlation of 0.73. The method has been implemented as part of PRODIGY.^18^

Other recent studies from Yang and collaborators have highlighted the importance of using interface descriptors to train an artificial neural network (ANN) to predict protein–protein binding affinity.^19,20^ They have generated a variety of machine learning (ML) and deep learning (DL) models. Their model is not general, as it requires the use of one distinct model for predicting binding affinities of antibody–antigens and another for protein–protein complexes.

In agreement with the importance of interfacial properties to predict affinities, we have also recently trained an ANN based on 14 UMAP-reduced Rosetta-calculated descriptors, where interface area, shape complementarity, solvent-accessible solvent area, and number of residues on the interface were listed among the most important features.^21^ We used the 81 protein complexes from PRODIGY for training. Validation was performed using PDBbind and a structural database containing all nanobody complexes available in the PDB. Prediction yielded an RMSE of ca. 2.5 kcal/mol and Pearson’s correlation of 0.53.^21^ Overall, these previous attempts using structure-based properties to train the ML/DL algorithm have not been able to provide a model that is both general and can accurately predict the absolute binding affinities/free energies of protein–protein association. In the present work, we aim to address the limitations of existing methods by developing a generalizable and accurate machine learning model for predicting the absolute binding affinities of protein–protein interactions. For this, a tailored extensive Rosetta^22^ descriptors list from hundreds of protein–protein structure complexes was used to train 10 ML algorithms. These algorithms were used as base learners to devise a super learner (SL) model.^23^ The SL, in turn, is an ensemble learning technique that combines predictions from multiple models (base learners) to achieve a better predictive performance than any single model. It leverages the strengths of various algorithms and reduces their weakness by creating a weighted combination linear regression of their outputs. Weights are calculated by non-negative least squares based on the Lawson–Hanson algorithm.^24,25^

We tested our SL model on 105 protein complexes (validation set) retrieved from the main protein databases (BENCHMARK-5.5,^26^ PDBbind,^27^ PRODIGY,^18^ and SKEMPI2^28^) and achieved an accuracy of 1.98 kcal/mol RMSE and a Pearson correlation of 0.70. Therefore, the SL model demonstrates improved accuracy and generalizability across diverse protein–protein complexes. The code is available as an open-source tool on GitHub (https://github.com/chavesejf/pbee). This work represents a significant step toward the efficient and scalable prediction of protein–protein binding affinities.

## Methods

### Data Sets

We have initially selected 603 X-ray resolved protein–protein complexes from the following databases: (i) PDBbind,^27^ (ii) PRODIGY,^18^ (iii) SKEMPI2,^28^ and (iv) protein–protein docking benchmark version 5.5^26^ that had no gaps or missing heavy atoms and with a maximum resolution of 2.5 Å. The data set was split into training and validation sets (more details in the Machine Learning section). An extensive manual inspection of protein–protein interface similarities was performed to ensure interface uniqueness and nonredundancy across training and validation sets. We have excluded mutants of the same protein–protein complex and complexes featuring similar three-dimensional interfaces across data sets, aiming to prevent data leaking. The final data set was comprised of 532 protein–protein complexes (split into 427 protein–protein complexes in the training set and 105 in the validation set). The number of protein–protein complexes from each database used in this work is shown in Table 1. It is important to highlight that PDBbind contains the largest number of protein–protein complexes, which in turn included complexes from the other data sets. The experimental affinity data for these protein–protein complexes were obtained from the databases themselves, and if necessary, the experimental binding free energy () was calculated from the dissociation constant (*K*_D_) based on the following relationship: , where *R* is the ideal gas constant, and *T* is the temperature in Kelvin (here considered as 298.15 K for all cases).

**Table 1:** Size of Nonredundant Data Set of Protein–Protein Complexes Used to Train and Validate ML Models

Database	Training set #	Validation set #
PDBbind^a^	366	90
PRODIGY^b^	2	-
SKEMPI2^c^	14	7
Benchmark 5.5^d^	45	8
Total	427	105

a http://pdbbind.org.cn.

b http://wenmr.science.uu.nl/prodigy/dataset.

c http://life.bsc.es/pid/skempi2.

d http://zlab.wenglab.org/benchmark.

### Structure Preprocessing

The preprocessing pipeline consisted of the following steps: (i) search for gaps in the backbone, (ii) add missing hydrogens, (iii) identify structural ion atoms such as Mg^2+^, Ca^2+^, Na^+^, Cl^–^, Fe^2+/3+^, K^+^, and Zn^2+^ with a Euclidean distance of less than 2.0 Å from any protein atom, and (iv) eliminate any other heteroatom types (e.g., nonstructural ion atoms, water molecules, small ligands, cofactors, and sugar moieties from glycosylation).

### Structure Postprocessing

#### Geometry Optimization

The resulting geometries (preprocessed structures) were optimized using the Rosetta software according to the following protocol: (i) a first optimization of the side chains with the backbone held fixed; (ii) a second optimization for all atoms, both using Rosetta’s standard minimization algorithm, adjusted to 50,000 steps and a convergence threshold of 10^–4^ Rosetta energy units; (iii) flipping histidine, asparagine, and glutamine residues were probed during hydrogen placement optimization, as well as sampling χ_1_ and χ_2_ rotamer angles for all residues. The BETA_NOV16 potentials^16,29^ were employed for scoring geometry optimization.

#### Descriptors of Protein–Protein Association

Protein–protein interface properties were computed using the *InteractionEnergyMetric*, *ContactMolecularSurface*, *InterfaceHoles*, and *InterfaceAnalyzerMover* applications^29^ within Rosetta.^18^ The *auto_setup_metals* parameter was activated if ions were detected in the structures. All properties were calculated using the BETA_NOV16 potentials.^16,29^ The final data set was comprised of 52 features (Table S1) for each of the 532 structures along with their experimental . (Unsupervised and supervised methods such as UMAP, PCA, LDA, and SHAP were used to attempt to reduce dimensionality. Final performance was poor in all cases. Data not shown for conciseness.)

### Machine Learning

**Figure 1:**
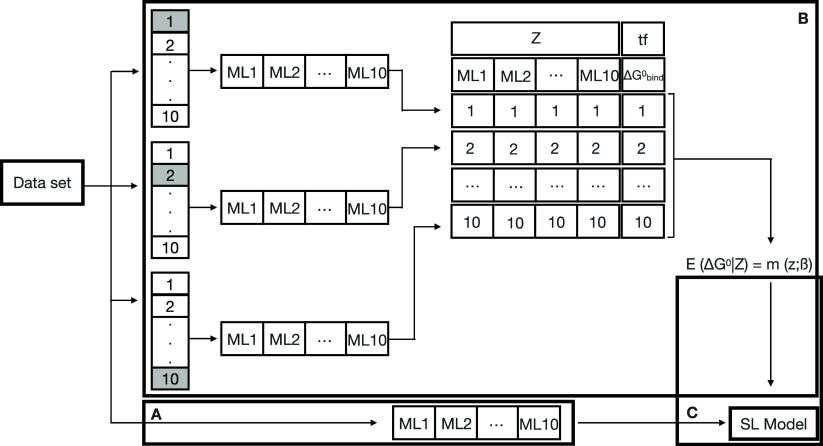
Schematic representation of the SL pipeline, which undergoes three steps. (A) The full training set is used to train 10 different ML algorithms, and (B) it was split into 10 subsets via K-fold. The subsets were trained by each ML algorithm and evaluated using k-fold cross-validation. All out-of-fold predictions were stored as a covariance matrix, and the model was fit to the full subset and stored. A meta-model (*m*(*z*;β)) was generated by estimating (*E*(Δ*G*^0^|*Z*)) the target function (Δ*G*^0^, or specifically here ) in the validation block on the corresponding training block candidate learner, where *Z* is the covariate vector. (C) A SL model is generated by combining predictions from each candidate using a weighted combination linear regression of their outputs of the full data set and the metamodel (*m*(*z*;β)).

We have coded a super learner (SL) model that combines 10 different ML algorithms: AdaBoost, bagging, decision tree, elastic net, extra trees, KNeighbors, linear regression, random forest, support vector regressor, and XGBoost. A schematic illustration of the super learner model can be viewed in Figure 1. The data set was split into 80% training set and 20% validation set, where a Gaussian distribution of the experimental binding free energies was present in both sets, therefore ensuring that the model was trained on the entire target property interval. The validation set was set apart and not used in the training (external validation). K-fold split the training set into 10 subsets, which were used to train all algorithms individually and a meta model was generated. The training set was also used to train each ML model individually using the same parameters for each case and the same K-fold cross-validation scheme. Extensive hyperparameter tuning was applied prior to defining the training engine. The number of iterations was set to 1,000,000 for elastic networks and 1,000 for bagging, random forest, and extra trees. The objective was set to square deviation error for extreme gradient boosting. The coefficient of determination (*R*^2^) was used as a loss function metric in all cases. The remaining parameters were set to default. The meta model was combined with the training results of each algorithm individually using the entire training set to generate the SL engine. Prediction is carried out using the test set, and accuracy was assessed by root-mean-square error (RMSE) and Pearson correlation (*R*). The remaining properties are fit to the individual base models, which are stacked to be used with the SL engine. The  is then computed for all individual ML and SL models.

### The PBEE Software

**Figure 2:**
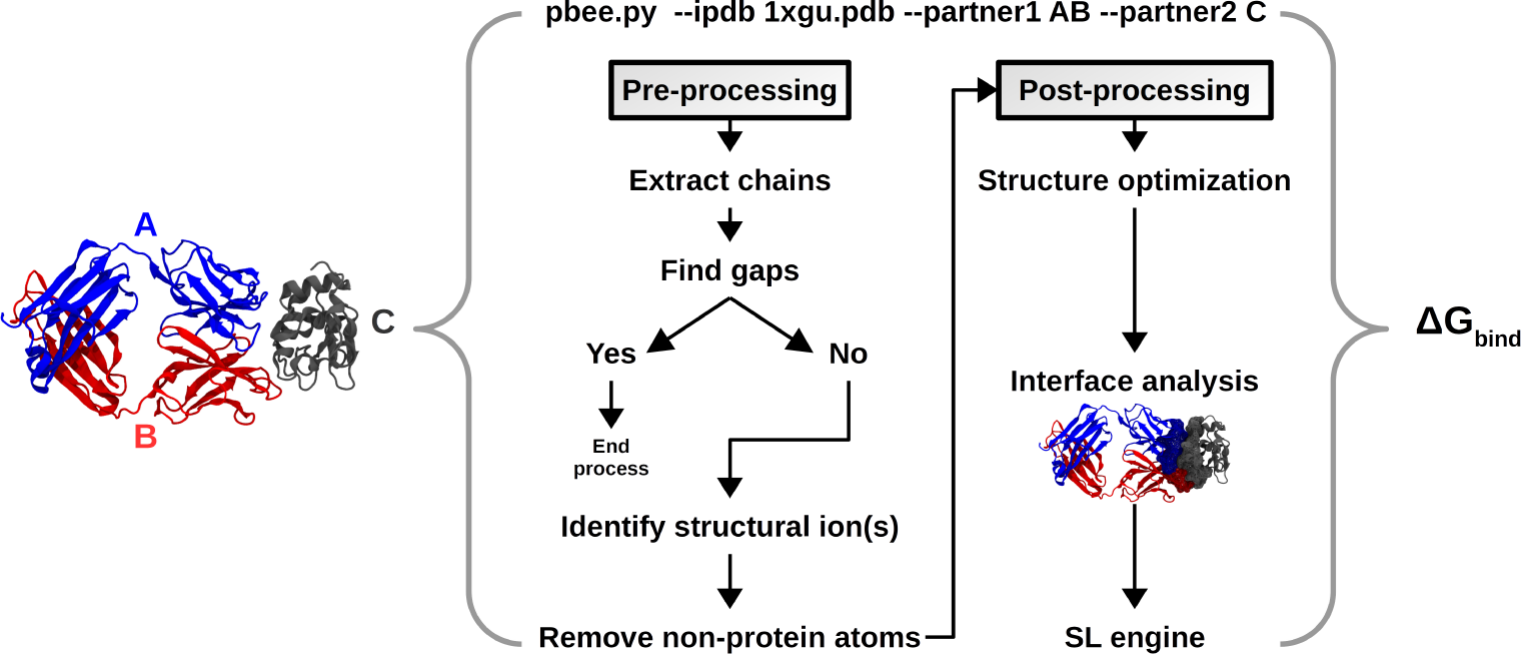
PBEE flowchart for the protein–protein complex structure PDB ID 1XGU, corresponding to antibody (HyHEL-63) that binds to lysozyme C (PDB 1XGU). Proteins are represented in cartoon model, where the antibody heavy and light chains are shown as chains “A″ and “B″ and color-coded in blue and red, respectively, while Lysozyme C is shown as “C″ and color-coded as dark gray. The pipeline works as follows: (i) the argument --ipdb receives the structure of the complex(es) in PDB format; (ii) the arguments --partner1 and --partner2 receive the chain IDs of the binding partners (it is worth noting that chains are renamed in order of appearance in the PDB file to A, B, C and so on); and (iii) PBEE runs the pre- and postprocessing stages and returns the free energy of binding of the complex in kcal/mol and corresponding dissociation constant, calculated by the SL model. The user can also choose to print predictions from any of the individual ML methods. In addition, we also deployed the PBEE code as a Google Colab notebook. It is designed as an alternative to enhance user accessibility, as there is no need for local installations, ensuring end user convenience with the same standalone features. The application can be used on any device. The code and instructions of how to use are available at: https://colab.research.google.com/drive/1lu1dC0yRltKK_Wp-gF26oHcZSCiHaI8b.

A Python pipeline called PBEE (protein binding energy estimator) was developed to easily calculate the absolute binding free energies from the coordinates of protein–protein complexes in the PDB format. The PBEE workflow, as well as an example of how to use it, as the command line, is shown in Figure 2. The software can be easily installed and run locally. It was built to run on a premade virtual environment, containing all dependencies needed to run the PBEE pipeline described in Figure 2. It uses PyRosetta to calculate the Rosetta descriptors and therefore bypasses the need of installing the Rosetta software.

## Results and Discussion

**Figure 3:**
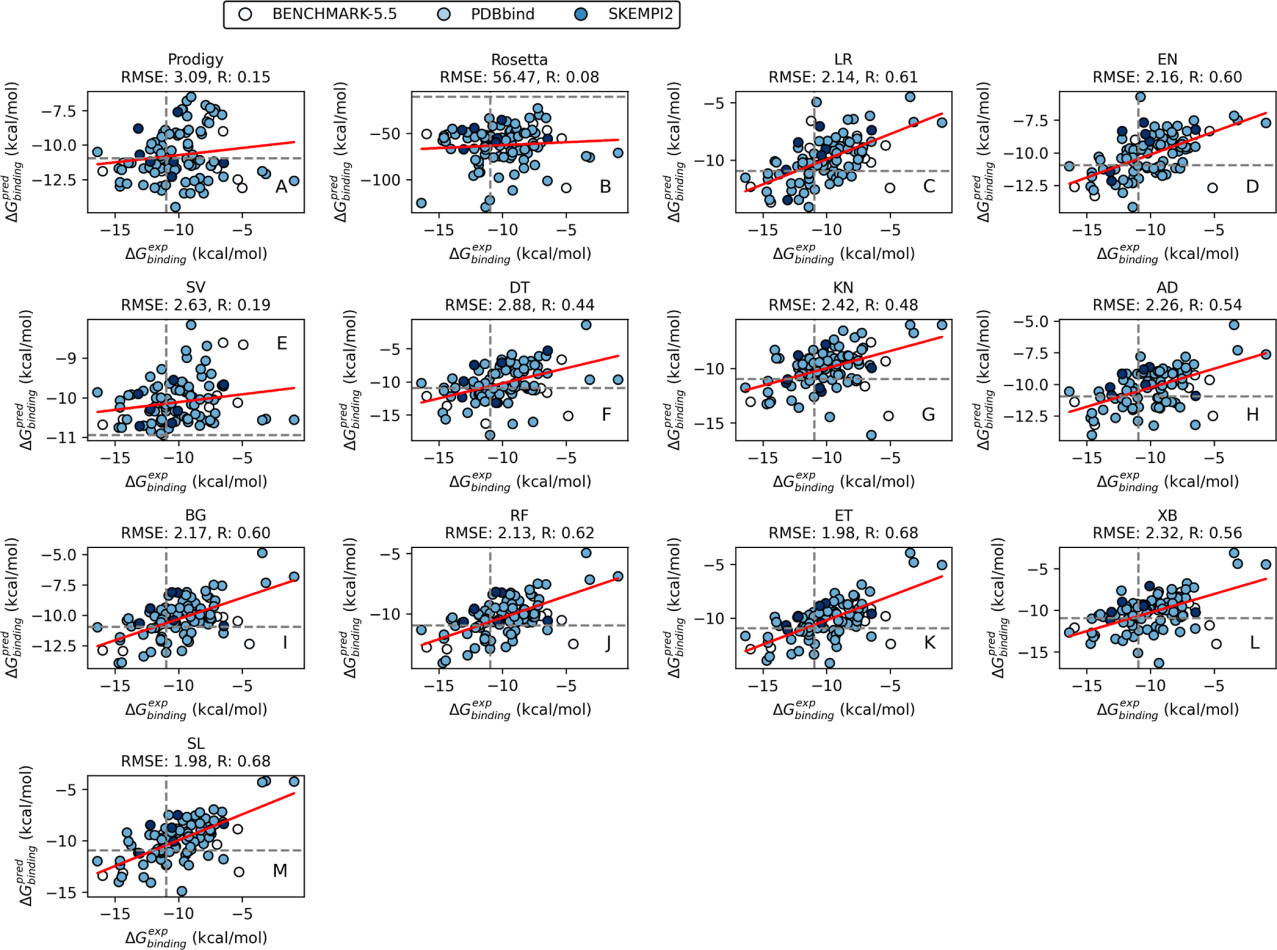
Regressions on the validation set using PRODIGY, Rosetta, and the trained ML and SL models (RMSE: root-mean-square error; *R*: Pearson correlation). Each point represents a protein-protein complex, and they are color-coded by the dataset they were collected (clear: Benchmark-5.5; light blue: PDBbind; blue: SKEMPI2). the database the protein–protein complexes were collected.

**Figure 4:**
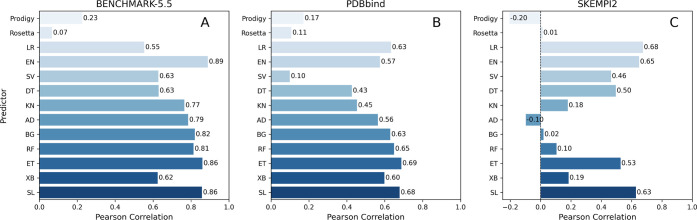
Comparison of PBEE prediction accuracy by ML method to Rosetta and PRODIGY, for the three protein–protein complex data sets.

We have assessed PBEE’s accuracy by the ML method, considering the protein–protein complexes included in the test set (Figure 3), but also its performance by database (Figure 4). Corresponding PRODIGY^18^ and Rosetta^18^ predictions were used for comparison. While the former is arguably considered the best performer, it is also important to evaluate the latter, as Rosetta has been used to generate the descriptors used in this study. The correlation between the calculated  by Rosetta is shown in Figure 3. The lack of correlation is not a surprise, as the Rosetta software was not specifically developed to predict binding free energies of protein–protein complexes. The use of any ML model represents an improvement over Rosetta’s BETA_NOV16^16,29^ (Figure 3). PRODIGY^18^ also represents an improvement over Rosetta’s prediction. As described in the literature, it performs extremely well on its own set of structures, but its prediction lacks the consistency of our SL model when other data sets are considered. When considering all structures in the four data sets, PRODIGY^18^ performance decreases significantly (Figure 4). Similarly, Laan et al. have also noticed that PRODIGY performance is compromised when a large data set is used.^23^ The authors developed the ProBAN method for predicting binding affinity in protein–protein complexes based on a deep convolutional neural network.^30^ The model was trained considering 3959 protein–protein complexes and evaluated using an external test for the PPI-Affinity service (82 complexes). Its prediction accuracy peaked at a Pearson correlation of 0.55 and an RMSE of 1.75 kcal/mol. In addition, in this study, PRODIGY also obtained a poor Pearson correlation for one of the data sets (*R* = 0.28).

In this study, SL and ET rank highest and tie in prediction accuracy when data sets are combined (Figure 3). While tree-based methods are well-known for bias reduction, SL creates an optimal weighted average of several ML models, as demonstrated by Laan et al. in their work on ensemble learning.^23^ Our data confirms that SL is as accurate as the best ML prediction algorithms. It achieves RMSE equal to 1.98 kcal/mol and *R* equal to 0.70 (Figure 3). However, the advantage of the SL model over individual models is shown when considering the different data sets (Figure 4). PBEE’s SL achieves a consistent level of accuracy, scoring higher among the best predictions regardless of the database. Therefore, given its robustness, the SL is set as the default in the PBEE pipeline, and it is the recommended option. Nevertheless, the user can select a different method to be used for  prediction for benchmarking purposes and/or ML method comparison.

**Figure 5:**
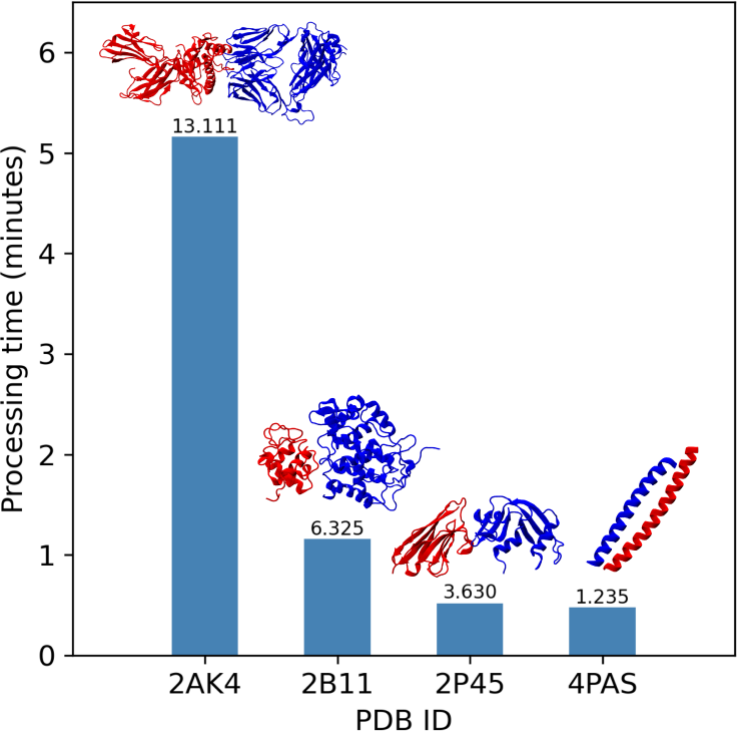
PBEE processing time as a function of the number of atoms in the protein–protein complex. Benchmark was conducted on a 13th generation Intel core i5-13450HX with 23 GM RAM. Numbers on top of the bars correspond to the number of atoms of the complexes.

Increasing PBEE’s overall accuracy faces some challenges. We are currently limited by the number of high-resolution structures, capping the size of the training data set to a few hundred structures. In addition, binding data come from different experimental conditions and techniques (e.g., SPR, BLI, ITC), and their intrinsic standard deviation errors are typically between 1% and 5%. Nevertheless, PBEE’s accuracy can be considered acceptable for high-throughput applications aiming for speeding up time to solution and saving laboratory costs. It represents an attractive strategy to triage the thousands or millions of binding decoys generated by structure-based computational protein design methods (e.g., Rosetta,^22^ FoldX,^31,32^ RFdiffusion^33^), as it can rapidly compute their association binding free energies. Benchmarks show that PBEE’s speed depends on the number of atoms. Most complexes take less than 2 min in a modern-day desktop computer (Figure 5), while large complexes can take over 5 min to compute. Nevertheless, as predictions for each protein–protein complex are independent, it will scale with the number of cores available.

**Figure 6:**
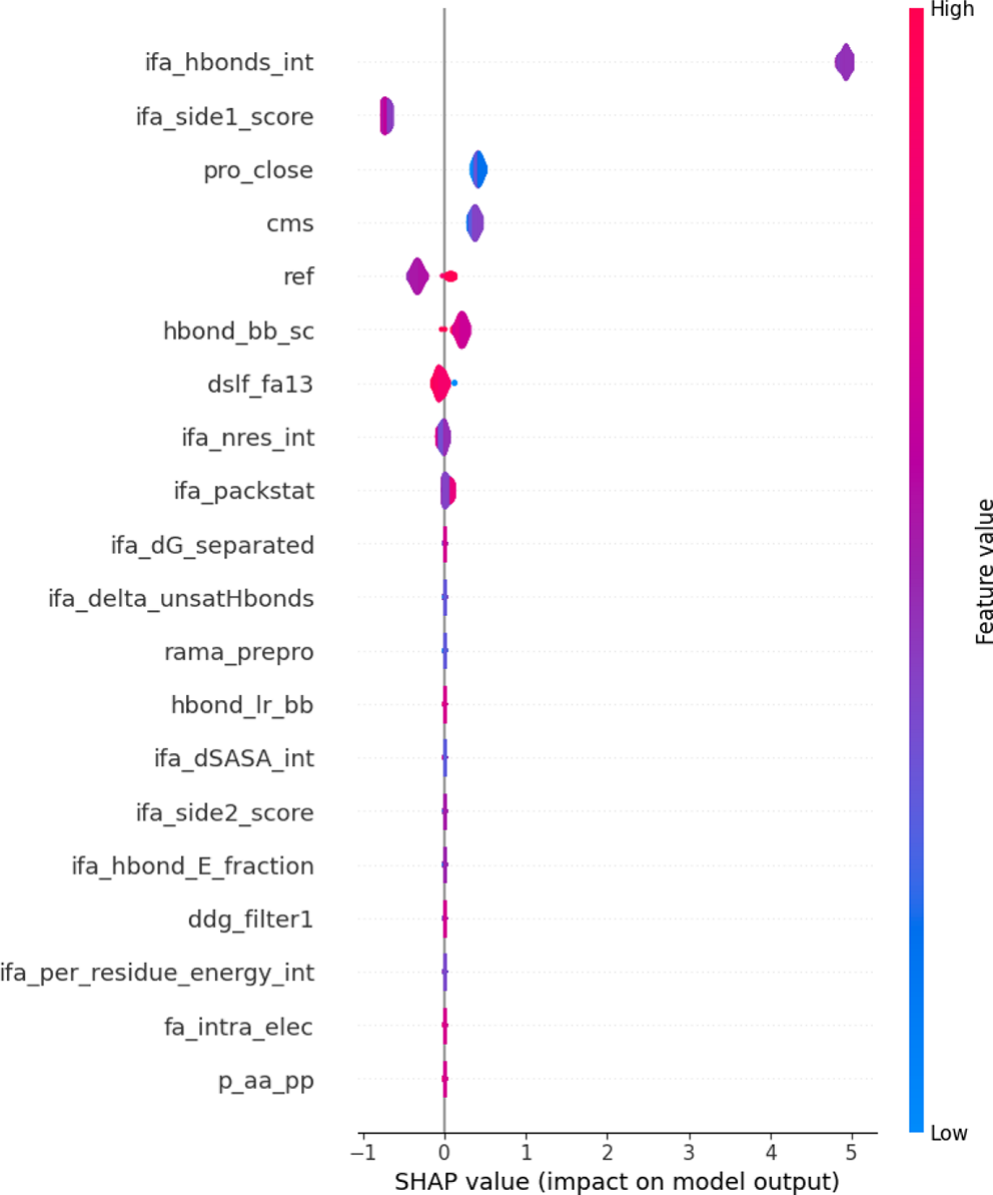
Feature importance is based on SHAP-values. The violin plots employ color gradients to convey insights. Shades of red signify higher values, indicating impactful features (Rosetta descriptors shown on the *y* axis) associated with the outcome (). In contrast, shades of blue represent lower values, highlighting features that influence the result differently. (Description of the Rosetta descriptors is available in Table S3.)

Moreover, to improve the interpretability of the SL model and understand the impact of each feature on its predictions, we have used SHAP (Shapley Additive exPlanations)^34^ (method details are available n the Supporting Information) to develop a model explainer for the SL. Figure 6 shows the 20 descriptors having the highest contribution to the prediction of the  according to the SL model. It revealed that quantities related to the protein–protein interface (such as the contact molecular surface (cms) and those calculated by the InterfaceAnalyzer routine, shown by the ifa prefix) exert a significant influence on the model’s outcome.

## Final Considerations

We have explored 10 different ML models for the prediction of protein–protein binding affinities and combined them then into a SL model. The SL engine was implemented into software named PBEE, which is available to download and use via GitHub. The prediction performance of PBEE is in pair with the most accurate methods available, regardless of computational requirements. It also does not require nontrivial setup, often associated with enhanced sampling techniques. While a few other models for the same purpose are available in the literature to date, we have used the most comprehensive, diverse, unbiased, and least redundant data set. In addition, validation was carried out on an external, randomly selected, data set of protein–protein complexes. Moreover, our SL engine implemented as PBEE accelerates high-throughput protein–protein binding affinity calculations, making it valuable for a myriad of biotechnological applications such as hit identification, pathogen immune evasion upon antigen mutations, evaluation of antibody therapy efficacy, and protein design endeavors in general. In addition, to date, PBEE provides unprecedented accuracy when compared to other ML/DL methods available for the same purpose.

## Data Availability

Data supporting the findings of this study are available within the electronic Supporting Information. A Python pipeline application is available to download and use from the GitHub link: https://github.com/chavesejf/PBEE. In addition, PBEE is also available as the Google Colab notebook at the following link: https://colab.research.google.com/drive/1lu1dC0yRltKK_Wp-gF26oHcZSCiHaI8b.

## Supporting Information Available

The list of structures from the Protein Data Bank (PDB) used in this work; the list of scoring functions and Rosetta descriptors used to train and validate the models; the RosettaScript used to calculate the descriptors; and the theoretical description of SHAP (PDF)

## Abbreviations

binding free energy
ANN: artificial neural network
DL: deep learning
LDA: linear discriminant analysis
ML: machine learning
PDB: protein data bank
PBEE: protein binding energy estimator
PCA: principal component analysis
R: Pearson correlation
RMSE: root-mean-square error
SHAP: Shapley Additive exPlanations
RFdiffusion: Rosetta fold diffusion
UMAP: uniform manifold approximation and projection
