# Supplementary material for: Estimating Absolute Protein–Protein Binding Free Energies by a Super Learner Model
Source: J Chem Inf Model. 2025 Feb 20;65(5):2602–9. doi: 10.1021/acs.jcim.4c01641 (PMC11898044; doi:10.1021/acs.jcim.4c01641)
Supplement: Supplementary file 1 — ci4c01641_si_001.pdf [file ci4c01641_si_001.pdf]

# SUPPORTING INFORMATION

# Estimating the Absolute Protein-Protein Binding Free Energy by a Super Learner Model

Elton J. F. Chaves[a], João Sartori[b], Whendel M. Santos[c], Carlos H. B. Cruz[d], Emmanuel N. Mhours[e], Manassés F. Nascimento-Filho[d], Matheus V. F. Ferraz[f], Roberto D. Lins[a,c,*]

[a]Aggeu Magalhães Institute, Oswaldo Cruz Foundation, Recife, Brazil., [b]Laboratory for Applied Genomics and Bio-Innovations, Oswaldo Cruz Foundation, Rio de Janeiro, Brazil, [c]Department of Fundamental Chemistry, Federal University of Pernambuco, Recife, Brazil. [d]Institute of Structural and Molecular Biology, University College London, UK. [e]Department of Computer Science, Princeton University, Princeton, USA. [f]NEC OncoImmunity, AS, Oslo, Norway

## ELECTRONIC SUPPLEMENTARY INFORMATION

**Table S1**. List of structures from the protein data bank (PDB) used in this work to train the machine learning methods, and the corresponding experimental binding free energy……………………..……………………….1

**Table S2**. List of structures from the protein data bank (PDB) used in this work used to validate (external data set) the machine learning methods, and the corresponding experimental binding free energy …………..…..12

**Table S3.** List of scoring functions and descriptors used to train the super model……………………………16

Theoretical description of SHAP (SHapley Additive exPlanations)………………………………………..18

**Table S1.** List of structures from the protein data bank (PDB) used in this work to train the machine learning methods, and the corresponding experimental binding free energy.

| | Database | PDB ID | Partner1 | Partner2 | ΔG$_{bind}$ | Description |
|---|---|---|---|---|---|---|
| 1 | BENCHMARK-5.5 | 3G6D | HL | A | -14.64 | Crystal structure of the complex between cnto607 fab and il-13 |
| 2 | BENCHMARK-5.5 | 2HRK | A | B | -10.98 | Structural basis of yeast aminoacyl-trna synthetase complex formation |
| 3 | BENCHMARK-5.5 | 1GRN | A | B | -8.75 | Crystal structure of the cdc42/cdc42gap/alf3 complex. |
| 4 | BENCHMARK-5.5 | 1DQJ | AB | C | -11.67 | Crystal structure of the anti-lysozyme antibody hyhel-63 complexed |
| 5 | BENCHMARK-5.5 | 1M10 | A | B | -11.2 | Crystal structure of the complex of glycoprotein ib alpha and the von |
| 6 | BENCHMARK-5.5 | 1FFW | A | B | -8.1 | Chey-binding domain of chea in complex with chey with a bound imido |
| 7 | BENCHMARK-5.5 | 3CPH | G | A | -8.8 | Crystal structure of sec4 in complex with rab-gdi |
| 8 | BENCHMARK-5.5 | 1B6C | A | B | -8.9 | Crystal structure of the cytoplasmic domain of the type i tgf-beta |
| 9 | BENCHMARK-5.5 | 1EZU | C | AB | -13.8 | Ecotin y69f d70p bound to d102n trypsin |
| 10 | BENCHMARK-5.5 | 1FQJ | A | B | -9.8 | Crystal structure of the heterotrimeric complex of the rgs domain of |
| 11 | BENCHMARK-5.5 | 2JEL | HL | P | -11.62 | Jel42 fab/hpr complex |
| 12 | BENCHMARK-5.5 | 1AY7 | A | B | -8.19 | Ribonuclease sa complex with barstar |
| 13 | BENCHMARK-5.5 | 2UUY | A | B | -11.26 | Structure of a tick tryptase inhibitor in complex with |
| 14 | BENCHMARK-5.5 | 3D5S | A | C | -10.35 | Crystal structure of efb-c (r131a) / c3d complex |
| 15 | BENCHMARK-5.5 | 2A9K | A | B | -9.85 | Crystal structure of the c3bot-nad-rala complex reveals a novel type |
| 16 | BENCHMARK-5.5 | 2X9A | D | C | -7.31 | Crystal structure of g3p from phage if1 in complex with its |
| 17 | BENCHMARK-5.5 | 2OUL | A | B | -11.96 | The structure of chagasin in complex with a cysteine protease |
| 18 | BENCHMARK-5.5 | 1EAW | A | B | -14.05 | Crystal structure of the mtsp1 (matriptase)-bpti (aprotinin) complex |
| 19 | BENCHMARK-5.5 | 1XD3 | A | B | -8.9 | Crystal structure of uchl3-ubvme complex |
| 20 | BENCHMARK-5.5 | 1R6Q | A | C | -8.8 | Clpns with fragments |
| 21 | BENCHMARK-5.5 | 1GPW | A | B | -10.53 | Structural evidence for ammonia tunneling across the (beta/alpha)8 |
| 22 | BENCHMARK-5.5 | 1AKJ | AB | ED | -5.3 | Complex of the human mhc class i glycoprotein hla-a2 and the t cell |
| 23 | BENCHMARK-5.5 | 1DFJ | E | I | -18.05 | Ribonuclease inhibitor complexed with ribonuclease a |
| 24 | BENCHMARK-5.5 | 1FLE | E | I | -12.3 | Crystal structure of elafin complexed with porcine pancreatic elastase |
| 25 | BENCHMARK-5.5 | 1XQS | A | C | -7.1 | Crystal structure of the hspbp1 core domain complexed with the |
| 26 | BENCHMARK-5.5 | 1LFD | B | A | -7.8 | Crystal structure of the active ras protein complexed with the ras- |
| 27 | BENCHMARK-5.5 | 2OOB | A | B | -5.7 | Crystal structure of the uba domain from cbl-b ubiquitin ligase in |
| 28 | BENCHMARK-5.5 | 1MQ8 | A | B | -7.5 | Crystal structure of alphal i domain in complex with icam-1 |
| 29 | BENCHMARK-5.5 | 1BVN | P | T | -15.07 | Pig pancreatic alpha-amylase in complex with the |
| 30 | BENCHMARK-5.5 | 2VIS | AB | C | -7.36 | Influenza virus hemagglutinin (escape) mutant with thr 131 replaced |
| 31 | BENCHMARK-5.5 | 1PXV | A | C | -12.97 | The staphostatin-staphopain complex: a forward binding inhibitor in |
| 32 | BENCHMARK-5.5 | 1J2J | A | B | -7.99 | Crystal structure of gga1 gat n-terminal region in complex with arf1 |
| 33 | BENCHMARK-5.5 | 2I25 | N | L | -12.3 | Crystal structure analysis of the nurse shark new antigen receptor |
| 34 | BENCHMARK-5.5 | 1KAC | A | B | -10.7 | Knob domain from adenovirus serotype 12 in complex with domain 1 of |
| 35 | BENCHMARK-5.5 | 1JTD | B | A | -14.41 | Crystal structure of beta-lactamase inhibitor protein-ii in complex |
| 36 | BENCHMARK-5.5 | 2O3B | A | B | -15.7 | Crystal structure complex of nuclease a (nuca) with intra-cellular |
| 37 | BENCHMARK-5.5 | 2J0T | A | D | -13.3 | Crystal structure of the catalytic domain of mmp-1 in complex with the |
| 38 | BENCHMARK-5.5 | 2BTF | A | P | -7.69 | The structure of crystalline profilin-beta-actin |
| 39 | BENCHMARK-5.5 | 1CLV | A | I | -12.28 | Yellow meal worm alpha-amylase in complex with the amaranth alpha- |
| 40 | BENCHMARK-5.5 | 2SIC | E | I | -13.84 | Refined crystal structure of the complex of subtilisin bpn' and |
| 41 | BENCHMARK-5.5 | 1PVH | A | B | -9.68 | Crystal structure of leukemia inhibitory factor in complex with gp130 |
| 42 | BENCHMARK-5.5 | 1KXQ | A | H | -11.54 | Camelid vhh domain in complex with porcine pancreatic alpha-amylase |
| 43 | BENCHMARK-5.5 | 1JIW | P | I | -15.55 | Crystal structure of the apr-aprin complex |
| 44 | BENCHMARK-5.5 | 1BVK | ED | F | -10.5 | Humanized anti-lysozyme fv complexed with lysozyme |
| 45 | BENCHMARK-5.5 | 1WQ1 | G | R | -6.51 | Ras-rasgap complex |
| 46 | PDBbind | 3DA7 | A | D | -9.39 | A conformationally strained circular permutant of barnase |
| 47 | PDBbind | 3ZEU | D | E | -8.92 | Structure of a salmonella typhimurium ygjd-yeaz heterodimer bound to |
| 48 | PDBbind | 1SYQ | A | B | -10.69 | Human vinculin head domain vh1 residues 1-258 in complex with human |
| 49 | PDBbind | 2OMW | A | B | -6.82 | Crystal structure of inla s192n y369s/mec1 complex |

| | | | | | | |
|---|---|---|---|---|---|---|
| 50 | PDBbind | 3WN7 | A | B | -11.1 | Crystal structure of keap1 in complex with the n-terminal region of |
| 51 | PDBbind | 6GUM | A | B | -8.08 | Structure of the a.thaliana e1 ufd domain in complex with e2 |
| 52 | PDBbind | 3G3B | A | B | -10.19 | Structure of a lamprey variable lymphocyte receptor mutant in complex |
| 53 | PDBbind | 1Y33 | E | I | -11.96 | Crystal structure of the complex of subtilisin bpn' with chymotrypsin |
| 54 | PDBbind | 1C1Y | A | B | -8.07 | Crystal structure of rap.Gmppnp In complex with the ras- |
| 55 | PDBbind | 5AYS | A | C | -12.68 | Crystal structure of saugi/hsv udg complex |
| 56 | PDBbind | 1F47 | B | A | -6.36 | The bacterial cell-division protein zipa and its interaction with an |
| 57 | PDBbind | 3H8K | A | B | -10.47 | Crystal structure of ube2g2 complxed with the g2br domain of gp78 at |
| 58 | PDBbind | 5M72 | A | B | -10.21 | Structure of the human srp68-72 protein-binding domain complex |
| 59 | PDBbind | 4W6Y | A | B | -12.01 | Co-complex structure of the lectin domain of f18 fimbrial adhesin fedf |
| 60 | PDBbind | 5VT9 | A | C | -10.91 | Myosin light chain 1 and myoa complex |
| 61 | PDBbind | 4EIG | A | B | -12.28 | Ca1698 camel antibody fragment in complex with dhfr |
| 62 | PDBbind | 2YGG | A | B | -10.5 | Complex of cambr and cam |
| 63 | PDBbind | 3OJ2 | A | C | -9.39 | Crystal structure of fgf1 complexed with the ectodomain of fgfr2b |
| 64 | PDBbind | 4JE4 | A | B | -10.71 | Crystal structure of monobody nsa1/shp2 n-sh2 domain complex |
| 65 | PDBbind | 5MTM | A | B | -11.13 | Monobody mb(lck_3) bound to lck-sh2 domain |
| 66 | PDBbind | 5MN2 | A | D | -7.62 | Cocrystal structure of fc gamma receptor iiia interacting with affimer |
| 67 | PDBbind | 4KJY | A | B | -14.17 | Complex of high-affinity sirp alpha variant fd6 with cd47 |
| 68 | PDBbind | 2OMX | A | B | -9.44 | Crystal structure of inla s192n g194s+s/hec1 complex |
| 69 | PDBbind | 3ZRZ | A | C | -14.36 | Crystal structure of the second and third fibronectin f1 modules in |
| 70 | PDBbind | 4AFQ | A | C | -11.76 | Human chymase - fynomer complex |
| 71 | PDBbind | 1SV0 | A | C | -10.85 | Crystal structure of yan-sam/mae-sam complex |
| 72 | PDBbind | 4XWJ | A | B | -11.39 | Histidine-containing phosphocarrier protein (hpr) and antisigma factor |
| 73 | PDBbind | 2P47 | A | B | -9.46 | Complex of a camelid single-domain vhh antibody fragment with rnase a |
| 74 | PDBbind | 2KSO | A | B | -7.21 | Epha2:ship2 sam:sam complex |
| 75 | PDBbind | 4Z9K | A | B | -11.81 | Ricin a chain bound to camelid nanobody (vhh2)(f5) |
| 76 | PDBbind | 4XKL | A | B | -6.01 | Crystal structure of ndp52 zf2 in complex with mono-ubiquitin |
| 77 | PDBbind | 5J28 | A | C | -9.16 | Ki67-pp1g (protein phosphatase 1 gamma isoform) holoenzyme complex |
| 78 | PDBbind | 5XOC | A | B | -6.95 | Crystal structure of human smad3-foxh1 complex |
| 79 | PDBbind | 3EQS | A | B | -11.58 | Crystal structure of human mdm2 in complex with a 12-mer peptide |
| 80 | PDBbind | 3P9W | A | B | -10.64 | Crystal structure of an engineered human autonomous vh domain in |
| 81 | PDBbind | 6JB8 | A | B | -13.49 | Crystal structure of nanobody d3-l11 in complex with hen egg-white |
| 82 | PDBbind | 3ONW | A | C | -11.78 | Structure of a g-alpha-i1 mutant with enhanced affinity for the rgs14 |
| 83 | PDBbind | 5YIS | A | C | -11.43 | Crystal structure of ankb lir/lc3b complex |
| 84 | PDBbind | 1U0S | Y | A | -9.06 | Chemotaxis kinase chea p2 domain in complex with response regulator |
| 85 | PDBbind | 3BZD | A | B | -5.48 | Manipulating the coupled folding and binding process drives |
| 86 | PDBbind | 5VKL | A | B | -9.48 | Spt6 tsh2-rpb1 1476-1500 ps1493 |
| 87 | PDBbind | 2YQ7 | A | B | -10.17 | Structure of bcl-xl bound to bimlock |
| 88 | PDBbind | 1CSO | E | I | -10.04 | Crystal structure of the omtky3 p1 variant omtky3-ile18i in |
| 89 | PDBbind | 5IP4 | A | E | -8.66 | X-ray structure of the c-terminal domain of human doublecortin |
| 90 | PDBbind | 3B08 | A | B | -6.5 | Crystal structure of the mouse hoil1-l-nzf in complex with linear di- |
| 91 | PDBbind | 2OMT | A | B | -9.55 | Crystal structure of inla g194s+s/hec1 complex |
| 92 | PDBbind | 1AN1 | E | I | -12.34 | Leech-derived tryptase inhibitor/trypsin complex |
| 93 | PDBbind | 3DVU | A | C | -9.14 | Crystal structure of the complex of murine gamma-herpesvirus 68 bcl-2 |
| 94 | PDBbind | 5XIU | A | B | -6.28 | Crystal structure of rnf168 udm2 in complex with lys63-linked |
| 95 | PDBbind | 4Y61 | A | B | -8.79 | Crystal structure of the complex between slitrk2 lrr1 and ptp delta |
| 96 | PDBbind | 6JJW | A | U | -11.03 | Crystal structure of kibra and ptpn14 complex |
| 97 | PDBbind | 5EP6 | A | B | -8.38 | The crystal structure of nap1 in complex with tbk1 |
| 98 | PDBbind | 5ML9 | A | B | -9.09 | Cocrystal structure of fc gamma receptor iiia interacting with affimer |
| 99 | PDBbind | 6NE4 | B | A | -12 | Designed repeat protein specifically in complex with fz7crd |
| 100 | PDBbind | 2VOH | A | B | -12.28 | Structure of mouse a1 bound to the bak bh3-domain |
| 101 | PDBbind | 5MTJ | A | B | -8.83 | Yes1-sh2 in complex with monobody mb(yes_1) |
| 102 | PDBbind | 5TDY | A | B | -10.09 | Structure of cofolded flifc:flign complex from thermotoga maritima |
| 103 | PDBbind | 4NOO | A | B | -11.33 | Molecular mechanism for self-protection against type vi secretion |

| | | | | | | |
|---|---|---|---|---|---|---|
| 104 | PDBbind | 5U4M | A | B | -12.84 | Rta-v1c7-g29r-no_salt |
| 105 | PDBbind | 3HCT | A | B | -7.95 | Crystal structure of traf6 in complex with ubc13 in the p1 space group |
| 106 | PDBbind | 1B3S | A | D | -13.55 | Structural response to mutation at a protein-protein interface |
| 107 | PDBbind | 2P43 | A | B | -10.42 | Complex of a camelid single-domain vhh antibody fragment with rnase a |
| 108 | PDBbind | 4BWQ | E | F | -6.42 | Crystal structure of u5-15kd in a complex with pqbp1 |
| 109 | PDBbind | 4DG4 | A | E | -7.6 | Human mesotrypsin-s39y complexed with bovine pancreatic trypsin |
| 110 | PDBbind | 2G45 | A | B | -7.57 | Co-crystal structure of znf ubp domain from the deubiquitinating |
| 111 | PDBbind | 5OYL | A | D | -6.99 | Vsv g cr2 |
| 112 | PDBbind | 3GJ8 | A | B | -7.53 | Crystal structure of human rangdp-nup153znf34 complex |
| 113 | PDBbind | 4YIQ | A | B | -7.77 | Structure of the ceacam6-ceacam8 heterodimer |
| 114 | PDBbind | 1SIB | E | I | -13.58 | Refined crystal structures of subtilisin novo in complex with wild- |
| 115 | PDBbind | 2WY8 | A | Q | -8.79 | Staphylococcus aureus complement subversion protein sbi-iv |
| 116 | PDBbind | 1T5Z | A | B | -6.11 | Crystal structure of the androgen receptor ligand binding |
| 117 | PDBbind | 5YIP | A | B | -11.5 | Crystal structure of ankg lir/gabarapl1 complex |
| 118 | PDBbind | 4DID | A | B | -7.12 | Crystal structure of salmonella effector n-terminal domain sopb in |
| 119 | PDBbind | 3RBB | A | B | -10.79 | Hiv-1 nef protein in complex with engineered hck sh3 domain |
| 120 | PDBbind | 1VEU | A | B | -10.77 | Crystal structure of the p14/mp1 complex at 2.15 a resolution |
| 121 | PDBbind | 3RZW | A | D | -9.78 | Crystal structure of the monobody ysmb-9 bound to human sumo1 |
| 122 | PDBbind | 1B2U | A | D | -9.55 | Structural response to mutation at a protein-protein interface |
| 123 | PDBbind | 2V8S | E | V | -6.35 | Vti1b habc domain - epsinr enth domain complex |
| 124 | PDBbind | 4AQE | A | B | -7.67 | Crystal structure of deafness associated mutant mouse cadherin-23 |
| 125 | PDBbind | 3GJ5 | A | B | -5.9 | Crystal structure of human rangdp-nup153znf4 complex |
| 126 | PDBbind | 5TVQ | A | B | -8.26 | Mouse tdp2 catalytic domain bound to sumo2 |
| 127 | PDBbind | 4RJF | A | B | -9.01 | Crystal structure of the human sliding clamp at 2.0 angstrom |
| 128 | PDBbind | 6FU9 | A | B | -11.36 | Complex of rice blast (magnaporthe oryzae) effector protein avr-pikd |
| 129 | PDBbind | 1P69 | A | B | -9.71 | Structural basis for variation in adenovirus affinity for the cellular |
| 130 | PDBbind | 3UYO | A | D | -7.05 | Crystal structure of monobody sh13/abl1 sh2 domain complex |
| 131 | PDBbind | 1X1X | A | D | -9.93 | Water-mediate interaction at aprotein-protein interface |
| 132 | PDBbind | 1OP9 | A | B | -12.49 | Complex of human lysozyme with camelid vhh hl6 antibody fragment |
| 133 | PDBbind | 3DXE | A | B | -8.84 | Crystal structure of the intracellular domain of human app (t668a |
| 134 | PDBbind | 1Y4D | E | I | -12.17 | Crystal structure of the complex of subtilisin bpn' with chymotrypsin |
| 135 | PDBbind | 3OJM | A | B | -9.39 | Crystal structure of fgf1 complexed with the ectodomain of fgfr2b |
| 136 | PDBbind | 4PC0 | A | C | -9.14 | Structure of the human rbap48-mta1(670-711) complex |
| 137 | PDBbind | 1AVA | A | C | -13.18 | Amy2/basi protein-protein complex from barley seed |
| 138 | PDBbind | 5G15 | A | B | -9.24 | Structure aurora a (122-403) bound to activating monobody mb1 and |
| 139 | PDBbind | 5EO9 | A | B | -8.77 | Crystal structure of the complex of dpr6 domain 1 bound to dip-alpha |
| 140 | PDBbind | 4PW9 | A | B | -6.64 | Crystal structure of the electron-transfer complex formed between a |
| 141 | PDBbind | 2W8B | A | H | -10.12 | Crystal structure of processed tolb in complex with pal |
| 142 | PDBbind | 1NF3 | A | C | -9.96 | Structure of cdc42 in a complex with the gtpase-binding domain of the |
| 143 | PDBbind | 6SAK | A | C | -10.24 | Structure of the otulincat c129a - snx27 pdz domain complex. |
| 144 | PDBbind | 4HRN | A | D | -11.57 | Structural basis for eliciting a cytotoxic effect in her2- |
| 145 | PDBbind | 2VLP | A | B | -16.73 | R54a mutant of e9 dnase domain in complex with im9 |
| 146 | PDBbind | 3NCB | A | B | -1.41 | A mutant human prolactin receptor antagonist h180a in complex with the |
| 147 | PDBbind | 6UYU | A | B | -7.77 | Crystal structure of k45-acetylated sumo1 in complex with |
| 148 | PDBbind | 3TL8 | A | B | -7.44 | The avrptob-bak1 complex reveals two structurally similar |
| 149 | PDBbind | 4U30 | A | X | -9.36 | Human mesotrypsin complexed with bikunin kunitz domain 2 |
| 150 | PDBbind | 3FP6 | E | I | -17.74 | Anionic trypsin in complex with bovine pancreatic trypsin inhibitor |
| 151 | PDBbind | 6UYS | A | B | -7.91 | Crystal structure of k37-acetylated sumo1 in complex with |
| 152 | PDBbind | 3C9A | B | D | -11.07 | High resolution crystal structure of argos bound to the egf domain of |
| 153 | PDBbind | 2JJS | A | C | -8.08 | Structure of human cd47 in complex with human signal regulatory |
| 154 | PDBbind | 2GOX | A | B | -12.41 | Crystal structure of efb-c / c3d complex |
| 155 | PDBbind | 2DVW | A | B | -9.78 | Structure of the oncoprotein gankyrin in complex with s6 atpase of the |
| 156 | PDBbind | 5AYR | A | B | -11.88 | The crystal structure of saugi/human udg complex |
| 157 | PDBbind | 1WRD | A | B | -4.62 | Crystal structure of tom1 gat domain in complex with ubiquitin |

| | | | | | | |
|---|---|---|---|---|---|---|
| 158 | PDBbind | 2SGP | E | I | -6.13 | Pro 18 variant of turkey ovomucoid inhibitor third domain complexed |
| 159 | PDBbind | 4JW2 | A | B | -11.5 | Selection of specific protein binders for pre-defined targets from an |
| 160 | PDBbind | 1X1W | A | D | -10.34 | Water-mediate interaction at aprotein-protein interface |
| 161 | PDBbind | 3OAK | A | D | -9.24 | Crystal structure of a spn1 (iws1)-spt6 complex |
| 162 | PDBbind | 3K6G | A | D | -10.62 | Crystal structure of rap1 and trf2 complex |
| 163 | PDBbind | 4H5S | A | B | -6.69 | Complex structure of necl-2 and crtam |
| 164 | PDBbind | 3QBQ | A | B | -9.06 | Crystal structure of extracellular domains of mouse rank-rankl complex |
| 165 | PDBbind | 6MOE | A | D | -11.9 | Monomeric darpin e2 complex with epor |
| 166 | PDBbind | 2XZE | B | R | -9.48 | Structural basis for amsh-escrt-iii chmp3 interaction |
| 167 | PDBbind | 1TM7 | E | I | -15.66 | Crystal structure of the complex of subtilisin bpn' with chymotrypsin |
| 168 | PDBbind | 4AYD | A | D | -8.3 | Structure of a complex between ccps 6 and 7 of human |
| 169 | PDBbind | 6J4O | A | B | -10.57 | Structural basis of tubulin detyrosination by vasohibins-svbp enzyme |
| 170 | PDBbind | 4J2L | A | C | -9.62 | Crystal structure of axh domain complexed with capicua |
| 171 | PDBbind | 2X2D | C | D | -7.11 | Acetyl-cypa:hiv-1 n-term capsid domain complex |
| 172 | PDBbind | 3GJ7 | A | B | -7.36 | Crystal structure of human rangdp-nup153znf12 complex |
| 173 | PDBbind | 5TL7 | A | B | -3.72 | Crystal structure of sars-cov papain-like protease in complex with c- |
| 174 | PDBbind | 1ZVY | A | B | -13.64 | Crystal structure of the vhh d3-l11 in complex with hen egg white |
| 175 | PDBbind | 4MQV | A | B | -7.55 | Crystal complex of rpa32c and smarcal1 n-terminus |
| 176 | PDBbind | 5DC4 | A | B | -10.17 | Crystal structure of monobody as25/abl1 sh2 domain complex crystal a |
| 177 | PDBbind | 2WWK | T | O | -8.04 | Crystal structure of the titin m10-obscurin like 1 ig f17r |
| 178 | PDBbind | 1J7D | A | B | -7.77 | Crystal structure of hmms2-hubc13 |
| 179 | PDBbind | 5H3J | A | B | -8.96 | Crystal structure of grasp domain of grasp55 complexed with the |
| 180 | PDBbind | 4K5B | D | A | -12.54 | Co-crystallization with conformation-specific designed ankyrin repeat |
| 181 | PDBbind | 1SGP | E | I | -11.51 | Ala 18 variant of turkey ovomucoid inhibitor third domain complexed |
| 182 | PDBbind | 5ELU | A | B | -8.71 | Isoform-specific inhibition of sumo-dependent protein-protein |
| 183 | PDBbind | 5AJJ | A | B | -7.49 | Crystal structure of variola virus virulence factor f1l in |
| 184 | PDBbind | 3KUC | A | B | -8.67 | Complex of rap1a(e30d/k31e)gdp with rafrbd(a85k/n71r) |
| 185 | PDBbind | 1OHZ | A | B | -12.22 | Cohesin-dockerin complex from the cellulosome of |
| 186 | PDBbind | 2WWX | A | B | -9.69 | Crystal structure of the sidm/drra(gef/gdf domain)-rab1 |
| 187 | PDBbind | 5ET1 | A | C | -9.51 | Crystal structure of myo3b-arb1 in complex with espin1-ar |
| 188 | PDBbind | 6AAF | A | B | -9.27 | Crystal structure of fission yeast atg8 complexed with the helical aim |
| 189 | PDBbind | 1TM4 | E | I | -13.49 | Crystal structure of the complex of subtilsin bpn'with chymotrypsin |
| 190 | PDBbind | 5UUL | A | B | -11.35 | Human bfl-1 in complex with puma bh3 |
| 191 | PDBbind | 4K1R | A | B | -6.81 | Crystal structure of schizosaccharomyces pombe sst2 catalytic domain |
| 192 | PDBbind | 1FSS | A | B | -12.86 | Acetylcholinesterase (e.c. 3.1.1.7) complexed with fasciculin-ii |
| 193 | PDBbind | 5NVM | A | B | -10.71 | Crystal structure of the human 4ehp-gigyf2 complex lacking the |
| 194 | PDBbind | 4IU3 | A | B | -10.48 | Cohesin-dockerin -x domain complex from ruminococcus flavefacience |
| 195 | PDBbind | 2B7C | A | B | -8.73 | Yeast guanine nucleotide exchange factor eef1balpha k205a mutant in |
| 196 | PDBbind | 4M0W | A | B | -7.06 | Crystal structure of sars-cov papain-like protease c112s mutant in |
| 197 | PDBbind | 1S1Q | A | B | -4.5 | Tsg101(uev) domain in complex with ubiquitin |
| 198 | PDBbind | 3MJH | A | B | -7.67 | Crystal structure of human rab5a in complex with the c2h2 zinc finger |
| 199 | PDBbind | 6JB2 | A | B | -8.91 | Crystal structure of nanobody d3-l11 mutant y102a in complex with hen |
| 200 | PDBbind | 2CCL | A | B | -12.13 | The s45a t46a mutant of the type i cohesin-dockerin |
| 201 | PDBbind | 3EBA | A | B | -11.77 | Cabhul6 fglw mutant (humanized) in complex with human lysozyme |
| 202 | PDBbind | 4C4P | A | B | -9.01 | Crystal structure of wild-type rab11 complexed to fip2 |
| 203 | PDBbind | 4AOR | A | D | -9.84 | Cationic trypsin in complex with the spinacia oleracea trypsin |
| 204 | PDBbind | 4MRT | C | A | -8.23 | Structure of the phosphopantetheine transferase sfp in complex with |
| 205 | PDBbind | 1VET | A | B | -10.77 | Crystal structure of p14/mp1 at 1.9 a resolution |
| 206 | PDBbind | 4C2A | A | B | -10.93 | Crystal structure of high-affinity von willebrand factor a1 domain |
| 207 | PDBbind | 3HTU | A | B | -7.84 | Crystal structure of the human vps25-vps20 subcomplex |
| 208 | PDBbind | 2OMU | A | B | -12.58 | Crystal structure of inla g194s+s y369s/hec1 complex |
| 209 | PDBbind | 2A78 | A | B | -9.85 | Crystal structure of the c3bot-rala complex reveals a novel type of |
| 210 | PDBbind | 4ZW2 | A | B | -11.34 | Crystal structure of the mouse voltage gated calcium channel beta |
| 211 | PDBbind | 1SBN | E | I | -14 | Refined crystal structures of subtilisin novo in complex with wild- |

| | | | | | | |
|---|---|---|---|---|---|---|
| 212 | PDBbind | 1Y48 | E | I | -11.96 | Crystal structure of the complex of subtilisin bpn' with chymotrypsin |
| 213 | PDBbind | 1UUZ | A | D | -10.37 | Ivy:a new family of protein |
| 214 | PDBbind | 1SGE | E | I | -8.56 | Glu 18 variant of turkey ovomucoid inhibitor third domain complexed |
| 215 | PDBbind | 1SGY | E | I | -12.8 | Tyr 18 variant of turkey ovomucoid inhibitor third domain complexed |
| 216 | PDBbind | 5YR0 | A | B | -6.98 | Structure of beclin1-uvrag coiled coil domain complex |
| 217 | PDBbind | 2W80 | A | D | -11.46 | Structure of a complex between neisseria meningitidis |
| 218 | PDBbind | 6FBX | A | B | -8.82 | Crystal structure of a zebra-fish pro-survival protein nrz:bad bh3 |
| 219 | PDBbind | 4OYD | A | B | -13.18 | Crystal structure of a computationally designed inhibitor of an |
| 220 | PDBbind | 2C7N | A | B | -7.09 | Human rabex-5 residues 1-74 in complex with ubiquitin |
| 221 | PDBbind | 1Y3D | E | I | -12.62 | Crystal structure of the complex of subtilisin bpn' with chymotrypsin |
| 222 | PDBbind | 5IMK | B | A | -8.28 | Nanobody targeting human vsig4 in spacegroup c2 |
| 223 | PDBbind | 5FVK | A | C | -7.84 | Crystal structure of vps4-vfa1 complex from s.cerevisiae at 1.66 a |
| 224 | PDBbind | 3U43 | A | B | -20.46 | Crystal structure of the colicin e2 dnase-im2 complex |
| 225 | PDBbind | 3D1M | A | D | -7.6 | Crystal structure of sonic hedgehog bound to the third fniii domain of |
| 226 | PDBbind | 4A94 | A | D | -15.43 | Structure of the carboxypeptidase inhibitor from nerita versicolor |
| 227 | PDBbind | 3KJ0 | A | B | -11.87 | Mcl-1 in complex with bim bh3 mutant i2dy |
| 228 | PDBbind | 2B10 | A | B | -6.82 | Crystal structure of the protein-protein complex between f82s |
| 229 | PDBbind | 4EQA | A | C | -11.75 | Crystal structure of pa1844 in complex with pa1845 from pseudomonas |
| 230 | PDBbind | 1LZW | A | B | -8.84 | Structural basis of clps-mediated switch in clpa substrate recognition |
| 231 | PDBbind | 3V1C | A | B | -10.26 | Crystal structure of de novo designed mid1-zinc |
| 232 | PDBbind | 1EMV | A | B | -18.58 | Crystal structure of colicin e9 dnase domain with its |
| 233 | PDBbind | 5O2T | A | B | -9.25 | Human kras in complex with darpin k27 |
| 234 | PDBbind | 6B12 | A | C | -11.04 | Structure of tne2 in complex with tni2 |
| 235 | PDBbind | 3GJ4 | A | B | -7.07 | Crystal structure of human rangdp-nup153znf3 complex |
| 236 | PDBbind | 3GJ3 | A | B | -5.88 | Crystal structure of human rangdp-nup153znf2 complex |
| 237 | PDBbind | 3ZU7 | A | B | -11.16 | Crystal structure of a designed selected ankyrin repeat protein in |
| 238 | PDBbind | 1T01 | A | B | -10.11 | Vinculin complexed with the vbs1 helix from talin |
| 239 | PDBbind | 2P48 | A | B | -9.46 | Complex of a camelid single-domain vhh antibody fragment with rnase a |
| 240 | PDBbind | 1TM3 | E | I | -14.63 | Crystal structure of the complex of subtilisin bpn' with chymotrypsin |
| 241 | PDBbind | 6DWF | A | G | -12.12 | Crystal structure of complex of bbki mutant l55r with bovine trypsin |
| 242 | PDBbind | 3WDG | A | B | -12.17 | Staphylococcus aureus udg / ugi complex |
| 243 | PDBbind | 2JJT | A | C | -8.08 | Structure of human cd47 in complex with human signal regulatory |
| 244 | PDBbind | 4GI3 | A | C | -12.51 | Crystal structure of greglin in complex with subtilisin |
| 245 | PDBbind | 2PSM | A | F | -14.36 | Crystal structure of interleukin 15 in complex with interleukin 15 |
| 246 | PDBbind | 1Y3B | E | I | -12.97 | Crystal structure of the complex of subtilisin bpn' with chymotrypsin |
| 247 | PDBbind | 1B27 | A | D | -19.1 | Structural response to mutation at a protein-protein interface |
| 248 | PDBbind | 4APX | A | B | -7.55 | Crystal structure of mouse cadherin-23 ec1-2 and protocadherin-15 |
| 249 | PDBbind | 1X1Y | A | D | -6.68 | Water-mediate interaction at aprotein-protein interface |
| 250 | PDBbind | 3WA0 | A | G | -7.61 | Crystal structure of merlin complexed with dcaf1/vprbp |
| 251 | PDBbind | 3LNZ | A | B | -12.69 | Crystal structure of human mdm2 with a 12-mer peptide inhibitor pmi |
| 252 | PDBbind | 2Z8V | A | D | -11.35 | Structure of an ignar-ama1 complex |
| 253 | PDBbind | 1OEY | A | J | -11.46 | Heterodimer of p40phox and p67phox pb1 domains from human nadph |
| 254 | PDBbind | 2G2U | A | B | -8.05 | Crystal structure of the shv-1 beta-lactamase/beta-lactamase inhibitor |
| 255 | PDBbind | 5JZE | B | A | -6.99 | Erve virus viral otu domain protease in complex with mouse isg15 |
| 256 | PDBbind | 3QBT | A | B | -8.25 | Crystal structure of ocrl1 540-678 in complex with rab8a:gppnhp |
| 257 | PDBbind | 1Y34 | E | I | -12.67 | Crystal structure of the complex of subtilisin bpn' with chymotrypsin |
| 258 | PDBbind | 3VV2 | A | B | -14.03 | Crystal structure of complex form between s324a-subtilisin and mutant |
| 259 | PDBbind | 1E6E | A | B | -8.27 | Adrenodoxin reductase/adrenodoxin complex of mitochondrial p450 |
| 260 | PDBbind | 3EQY | A | C | -11 | Crystal structure of human mdmx in complex with a 12-mer peptide |
| 261 | PDBbind | 6BX8 | A | B | -9.14 | Human mesotrypsin (prss3) complexed with tissue factor pathway |
| 262 | PDBbind | 3D5R | A | C | -10.53 | Crystal structure of efb-c (n138a) / c3d complex |
| 263 | PDBbind | 6E3I | A | B | -11.59 | Human bfl-1 in complex with the bfl-1-specific designed peptide srt.F4 |
| 264 | PDBbind | 5V5I | A | B | -8.64 | Otu protease of crimean congo hemorrhagic fever virus bound to |
| 265 | PDBbind | 2P1L | A | B | -7.69 | Structure of the bcl-xl:beclin 1 complex |

| | | | | | | |
|---|---|---|---|---|---|---|
| 266 | PDBbind | 1TM1 | E | I | -15.72 | Crystal structure of the complex of subtilisin bpn' with chymotrypsin |
| 267 | PDBbind | 1Y1K | E | I | -12.99 | Crystal structure of the complex of subtilisin bpn' with chymotrypsin |
| 268 | PDBbind | 6FUD | A | B | -9.74 | Complex of rice blast (magnaporthe oryzae) effector protein avr-pika |
| 269 | PDBbind | 1M9E | A | D | -5.7 | X-ray crystal structure of cyclophilin a/hiv-1 ca n-terminal domain |
| 270 | PDBbind | 6J9H | A | B | -10.06 | Crystal structure of svbp-vash1 complex |
| 271 | PDBbind | 1ZC3 | A | D | -10.06 | Crystal structure of the ral-binding domain of exo84 in complex with |
| 272 | PDBbind | 3QC8 | A | B | -6.75 | Crystal structure of faf1 ubx domain in complex with p97/vcp n domain |
| 273 | PDBbind | 4REY | A | B | -9.5 | Crystal structure of the grasp65-gm130 c-terminal peptide complex |
| 274 | PDBbind | 4Q5U | A | C | -16.37 | Structure of calmodulin bound to its recognition site from calcineurin |
| 275 | PDBbind | 6KBR | A | C | -15.08 | Crystal structure of human klk4 and spink2 derived klk4 inhibitor |
| 276 | PDBbind | 3GQI | A | B | -10.21 | Crystal structure of activated receptor tyrosine kinase in complex |
| 277 | PDBbind | 5W3X | A | B | -7.47 | Crystal structure of popp2 in complex with ip6 accoa and the wrky |
| 278 | PDBbind | 4MI8 | A | C | -7.99 | Crystal structure of the complex of murine gamma-herpesvirus 68 bcl-2 |
| 279 | PDBbind | 3VPJ | A | E | -11.67 | Crystal structure of type vi effector tse1 from pseudomonas aeruginosa |
| 280 | PDBbind | 5WUJ | A | B | -8.7 | Crystal structure of flif-flig complex from h. Pylori |
| 281 | PDBbind | 4D0G | A | C | -7.84 | Structure of rab14 in complex with rab-coupling protein (rcp) |
| 282 | PDBbind | 3SGB | E | I | -14.66 | Structure of the complex of streptomyces griseus protease b and the |
| 283 | PDBbind | 5DJU | B | A | -10.84 | Crystal structure of lov2 (c450a) domain in complex with zdk3 |
| 284 | PDBbind | 3DXD | A | B | -8.82 | Crystal structure of the intracellular domain of human app (t668e |
| 285 | PDBbind | 3FPU | A | B | -13.53 | The crystallographic structure of the complex between evasin-1 and |
| 286 | PDBbind | 3DXC | A | B | -9.08 | Crystal structure of the intracellular domain of human app in complex |
| 287 | PDBbind | 4LGP | A | B | -13.15 | Ricin a chain bound to camelid nanobody (vhh1) |
| 288 | PDBbind | 1VRK | A | B | -11.14 | The 1.9 angstrom structure of e84k-calmodulin rs20 peptide complex |
| 289 | PDBbind | 1TMG | E | I | -14.68 | Crystal structure of the complex of subtilisin bpn' with chymotrypsin |
| 290 | PDBbind | 4W6X | A | B | -11.3 | Co-complex structure of the lectin domain of f18 fimbrial adhesin fedf |
| 291 | PDBbind | 3VUX | A | G | -6.88 | Crystal structure of a20 zf7 in complex with linear ubiquitin form ii |
| 292 | PDBbind | 3VEP | X | D | -6.8 | Crystal structure of sigd4 in complex with its negative regulator rsda |
| 293 | PDBbind | 3T04 | A | D | -9.94 | Crystal structure of monobody 7c12/abl1 sh2 domain complex |
| 294 | PDBbind | 3KNB | A | B | -8.22 | Crystal structure of the titin c-terminus in complex with obscurin- |
| 295 | PDBbind | 4EKD | A | B | -10.44 | Structure of human regulator of g protein signaling 2 (rgs2) in |
| 296 | PDBbind | 6H46 | A | B | -10.26 | Human kras in complex with darpin k13 |
| 297 | PDBbind | 6GFI | A | E | -12.7 | Structure of human mesotrypsin in complex with appi variant |
| 298 | PDBbind | 5V5G | A | B | -10.98 | Otu protease of crimean congo hemorrhagic fever virus bound to |
| 299 | PDBbind | 3BTF | E | I | -10.91 | The crystal structures of the complexes between bovine beta-trypsin |
| 300 | PDBbind | 4EHQ | A | G | -8.03 | Crystal structure of calmodulin binding domain of orai1 in complex |
| 301 | PDBbind | 3M18 | A | B | -13.29 | Crystal structure of variable lymphocyte receptor vlra.R2.1 in complex |
| 302 | PDBbind | 2OIN | A | C | -8.58 | Crystal structure of hcv ns3-4a r155k muntant |
| 303 | PDBbind | 1SGN | E | I | -11.11 | Asn 18 variant of turkey ovomucoid inhibitor third domain complexed |
| 304 | PDBbind | 3FJU | A | B | -12 | Ascaris suum carboxypeptidase inhibitor in complex with human |
| 305 | PDBbind | 3FXD | A | B | -10.5 | Crystal structure of interacting domains of icmr and icmq |
| 306 | PDBbind | 6ASR | A | B | -6.58 | Rev1 ubm2 domain complex with ubiquitin |
| 307 | PDBbind | 1B2S | A | D | -13.38 | Structural response to mutation at a protein-protein interface |
| 308 | PDBbind | 2I26 | N | L | -10.95 | Crystal structure analysis of the nurse shark new antigen receptor |
| 309 | PDBbind | 5H9B | A | B | -8.42 | Drosophila camkii-wt in complex with a fragment of the eag potassium |
| 310 | PDBbind | 1SMF | E | I | -9.44 | Studies on an artificial trypsin inhibitor peptide derived from the |
| 311 | PDBbind | 4YN0 | A | B | -9.65 | Crystal structure of app e2 domain in complex with dr6 crd domain |
| 312 | PDBbind | 3G9W | A | D | -6.06 | Crystal structure of talin2 f2-f3 in complex with the integrin beta1d |
| 313 | PDBbind | 2J12 | A | B | -10.5 | Ad37 fibre head in complex with car d1 |
| 314 | PDBbind | 1TE1 | A | B | -11.55 | Crystal structure of family 11 xylanase in complex with inhibitor |
| 315 | PDBbind | 6AKM | A | B | -7.04 | Crystal structure of slmap-sike1 complex |
| 316 | PDBbind | 5MRV | A | C | -7.39 | Crystal structure of human carboxypeptidase o in complex with nvci |
| 317 | PDBbind | 1CT0 | E | I | -10.36 | Crystal structure of the omtky3 p1 variant omtky3-ser18i in |
| 318 | PDBbind | 2SGQ | E | I | -11.89 | Gln 18 variant of turkey ovomucoid inhibitor third domain complexed |
| 319 | PDBbind | 4Z80 | A | C | -10.81 | Crystal structure of toxoplasma gondii ama4 di-dii-egf1 in complex |

| | | | | | | |
|---|---|---|---|---|---|---|
| 320 | PDBbind | 5F5S | A | B | -10.45 | Crystal structure of the prp38-mfap1 complex of homo sapiens |
| 321 | PDBbind | 4ZI2 | A | C | -8.7 | Bart-like domain of bartl1/ccdc104 in complex with arl3fl bound to |
| 322 | PDBbind | 5EB1 | A | B | -9.35 | The yfib-yfir complex |
| 323 | PDBbind | 4BQD | A | C | -12.69 | Kd1 of human tfpi in complex with a synthetic peptide |
| 324 | PDBbind | 6F3Z | A | B | -8.72 | Complex of e. Coli lola and periplasmic domain of lolc |
| 325 | PDBbind | 4K5A | A | B | -10.9 | Co-crystallization with conformation-specific designed ankyrin repeat |
| 326 | PDBbind | 4I0C | A | D | -8.65 | The structure of the camelid antibody cabhul5 in complex with human |
| 327 | PDBbind | 5G1X | A | B | -6.71 | Crystal structure of aurora-a kinase in complex with n-myc |
| 328 | PDBbind | 4ZRK | A | E | -8.19 | Merlin-ferm and lats1 complex |
| 329 | PDBbind | 2WP3 | T | O | -8.32 | Crystal structure of the titin m10-obscurin like 1 ig |
| 330 | PDBbind | 2NU4 | E | I | -11.32 | Accommodation of positively-charged residues in a hydrophobic |
| 331 | PDBbind | 5N48 | A | B | -11.32 | Structure of anticalin n9b in complex with extra-domain b of human |
| 332 | PDBbind | 3TSR | A | E | -21.42 | X-ray structure of mouse ribonuclease inhibitor complexed with mouse |
| 333 | PDBbind | 6FUB | B | A | -10.28 | Complex of rice blast (magnaporthe oryzae) effector protein avr-pike |
| 334 | PDBbind | 4JS0 | A | B | -7.23 | Complex of cdc42 with the crib-pr domain of irsp53 |
| 335 | PDBbind | 6S8S | A | B | -8.71 | Extended structure of the human ddx6 c-terminal domain in complex with |
| 336 | PDBbind | 2J8X | A | B | -11.05 | Epstein-barr virus uracil-dna glycosylase in complex with ugi from |
| 337 | PDBbind | 5XCO | A | B | -10.98 | Crystal structure of human k-ras g12d mutant in complex with gdp and |
| 338 | PDBbind | 3P92 | A | E | -11.23 | Human mesotrypsin complexed with bovine pancreatic trypsin inhibitor |
| 339 | PDBbind | 5DJT | A | B | -10.6 | Crystal structure of lov2 (c450a) domain in complex with zdk2 |
| 340 | PDBbind | 5JW9 | A | B | -9.64 | The crystal structure of ell2 occludin domain and aff4 peptide |
| 341 | PDBbind | 6FHP | A | C | -9.98 | Daip in complex with a c-terminal fragment of thermolysin |
| 342 | PDBbind | 6DWH | D | J | -13.23 | Crystal structure of complex of bbki and bovine trypsin |
| 343 | PDBbind | 2VLO | A | B | -16.44 | K97a mutant of e9 dnase domain in complex with im9 |
| 344 | PDBbind | 2C7M | A | B | -7.09 | Human rabex-5 residues 1-74 in complex with ubiquitin |
| 345 | PDBbind | 5J4A | A | B | -9.74 | Cdia-ct toxin from burkholderia pseudomallei e479 in complex with |
| 346 | PDBbind | 1X1U | A | D | -10.75 | Water-mediate interaction at aprotein-protein interface |
| 347 | PDBbind | 3QQ8 | A | B | -7.95 | Crystal structure of p97-n in complex with faf1-ubx |
| 348 | PDBbind | 1SGQ | E | I | -9.52 | Gly 18 variant of turkey ovomucoid inhibitor third domain complexed |
| 349 | PDBbind | 2V5Q | A | D | -9.75 | Crystal structure of wild-type plk-1 kinase domain in complex with a |
| 350 | PDBbind | 3BS5 | A | B | -9.6 | Crystal structure of hcnk2-sam/dhyp-sam complex |
| 351 | PDBbind | 2NU2 | E | I | -11.14 | Accommodation of positively-charged residues in a hydrophobic |
| 352 | PDBbind | 3UL4 | A | B | -10.84 | Crystal structure of coh-olpa(cthe_3080)-doc918(cthe_0918) complex: a |
| 353 | PDBbind | 5IMM | B | A | -11.54 | Nanobody targeting mouse vsig4 in spacegroup p212121 |
| 354 | PDBbind | 3R85 | A | E | -5.98 | Crystal structure of human soul bh3 domain in complex with bcl-xl |
| 355 | PDBbind | 1YCS | A | B | -10.26 | P53-53bp2 complex |
| 356 | PDBbind | 1LW6 | E | I | -15.96 | Crystal structure of the complex of subtilisin bpn' with chymotrypsin |
| 357 | PDBbind | 5TZP | A | B | -10.26 | Crystal structure of fpv039:bik bh3 complex |
| 358 | PDBbind | 2D10 | A | E | -11.96 | Crystal structure of the radixin ferm domain complexed with the nherf- |
| 359 | PDBbind | 2VLQ | A | B | -14.52 | F86a mutant of e9 dnase domain in complex with im9 |
| 360 | PDBbind | 5KY4 | A | B | -6.82 | Mouse pofut1 in complex with mouse notch1 egf26 and gdp |
| 361 | PDBbind | 2R9P | A | E | -6.62 | Human mesotrypsin complexed with bovine pancreatic trypsin |
| 362 | PDBbind | 6IU7 | A | B | -12.34 | Crystal structure of importin-alpha1 bound to the 53bp1 nuclear |
| 363 | PDBbind | 3K2M | A | D | -11.13 | Crystal structure of monobody ha4/abl1 sh2 domain complex |
| 364 | PDBbind | 5TAR | A | B | -7.69 | Crystal structure of farnesylated and methylated kras4b in complex |
| 365 | PDBbind | 4DM6 | A | E | -7.95 | Crystal structure of rarb lbd homodimer in complex with ttnpb |
| 366 | PDBbind | 6NDZ | A | B | -13.23 | Designed repeat protein in complex with fz8 |
| 367 | PDBbind | 5INB | A | B | -9.43 | Repoman-pp1g (protein phosphatase 1 gamma isoform) holoenzyme complex |
| 368 | PDBbind | 2P42 | A | B | -10.42 | Complex of a camelid single-domain vhh antibody fragment with rnase a |
| 369 | PDBbind | 2OMZ | A | B | -8.73 | Crystal structure of inla y369a/hec1 complex |
| 370 | PDBbind | 4MIT | A | E | -9.21 | Crystal structure of e. Histolytica racc bound to the ehpak4 pbd |
| 371 | PDBbind | 4C7N | A | B | -12.28 | Crystal structure of the synthetic peptide im10 in complex |
| 372 | PDBbind | 3N0P | A | B | -3.13 | A mutant human prolactin receptor antagonist h30a in complex with the |
| 373 | PDBbind | 3QHY | A | B | -16.31 | Structural thermodynamic and kinetic analysis of the picomolar |

| | | | | | | |
|---|---|---|---|---|---|---|
| 374 | PDBbind | 2WH6 | A | B | -10.57 | Crystal structure of anti-apoptotic bhrf1 in complex with the bim bh3 |
| 375 | PDBbind | 1WQJ | B | I | -8.27 | Structural basis for the regulation of insulin-like growth factors |
| 376 | PDBbind | 5FZT | A | B | -5.89 | The crystal structure of r7r8 in complex with a dlc1 |
| 377 | PDBbind | 4NIQ | A | C | -7.23 | Crystal structure of vps4 mit-vfa1 mim2 |
| 378 | PDBbind | 4ML7 | A | B | -13.05 | Crystal structure of brucella abortus plic in complex with human |
| 379 | PDBbind | 3N4I | A | B | -12.22 | Crystal structure of the shv-1 d104e beta-lactamase/beta-lactamase |
| 380 | PDBbind | 4LLO | A | B | -6.66 | Structure of the eag domain-cnbhd complex of the mouse eag1 channel |
| 381 | PDBbind | 1SGD | E | I | -8.87 | Asp 18 variant of turkey ovomucoid inhibitor third domain complexed |
| 382 | PDBbind | 5WOS | A | B | -8.8 | Structural and functional insights into canarypox virus cnp058 |
| 383 | PDBbind | 2PTT | A | B | -7.36 | Structure of nk cell receptor 2b4 (cd244) bound to its ligand cd48 |
| 384 | PDBbind | 4UF1 | A | B | -7.03 | Deerpox virus dpv022 in complex with bak bh3 |
| 385 | PDBbind | 4H6J | A | B | -9.17 | Identification of cys 255 in hif-1 as a novel site for development of |
| 386 | PDBbind | 4BD9 | A | B | -10.24 | Structure of the complex between smci and human carboxypeptidase a4 |
| 387 | PDBbind | 5X4L | A | C | -7.68 | Crystal structure of the ubx domain of human ubxd7 in complex with p97 |
| 388 | PDBbind | 4AOQ | A | D | -9.13 | Cationic trypsin in complex with mutated spinacia oleracea trypsin |
| 389 | PDBbind | 2VLN | A | B | -16.06 | N75a mutant of e9 dnase domain in complex with im9 |
| 390 | PDBbind | 3IXS | A | B | -9.61 | Ring1b c-terminal domain/rybp c-terminal domain complex |
| 391 | PDBbind | 1CT2 | E | I | -11.3 | Crystal structure of the omtky3 p1 variant omtky3-thr18i in |
| 392 | PDBbind | 2VAY | A | B | -11.05 | Calmodulin complexed with cav1.1 iq peptide |
| 393 | PDBbind | 1KXV | A | C | -11.54 | Camelid vhh domains in complex with porcine pancreatic alpha-amylase |
| 394 | PDBbind | 4M5F | A | B | -14.83 | Complex structure of tse3-tsi3 |
| 395 | PDBbind | 5VKO | A | B | -11.46 | Spt6 tsh2-rpb1 1468-1500 pt1471 ps1493 |
| 396 | PDBbind | 3BK3 | A | C | -10.44 | Crystal structure of the complex of bmp-2 and the first von willebrand |
| 397 | PDBbind | 5GJK | A | B | -9.44 | Crystal structure of baf47 and baf155 complex |
| 398 | PDBbind | 1GUA | A | B | -10.09 | Human rap1a residues 1-167 double mutant (e30d;k31e) complexed with |
| 399 | PDBbind | 1T0P | A | B | -6.28 | Structural basis of icam recognition by integrin alpahlbeta2 revealed |
| 400 | PDBbind | 2V3B | A | B | -7.23 | Crystal structure of the electron transfer complex rubredoxin - |
| 401 | PDBbind | 6BXC | A | D | -10.76 | Crystal structure of n-terminal fragment of zebrafish toll-like |
| 402 | PDBbind | 3QHT | A | C | -9.67 | Crystal structure of the monobody ysmb-1 bound to yeast sumo |
| 403 | PDBbind | 2F4M | A | B | -9.8 | The mouse pngase-hr23 complex reveals a complete remodulation of the |
| 404 | PDBbind | 4ETW | A | B | -7.52 | Structure of the enzyme-acp substrate gatekeeper complex required for |
| 405 | PDBbind | 2B0Z | A | B | -5.45 | Crystal structure of the protein-protein complex between f82i |
| 406 | PDBbind | 2P4A | A | B | -13.29 | X-ray structure of a camelid affinity matured single-domain vhh |
| 407 | PDBbind | 3KJ2 | A | B | -11.87 | Mcl-1 in complex with bim bh3 mutant f4ae |
| 408 | PDBbind | 3P95 | A | E | -7.95 | Human mesotrypsin complexed with bovine pancreatic trypsin inhibitor |
| 409 | PDBbind | 4ZQU | A | B | -10.64 | Cdia-ct/cdii toxin and immunity complex from yersinia |
| 410 | PDBbind | 2QHO | A | B | -5.76 | Crystal structure of the uba domain from edd ubiquitin ligase in |
| 411 | PDBbind | 1RI8 | A | B | -11.65 | Crystal structure of the camelid single domain antibody 1d2l19 in |
| 412 | prodigy | 2VIR | AB | C | -12.27 | Influenza virus hemagglutinin complexed with a neutralizing |
| 413 | prodigy | 1CBW | ABC | D | -10.7 | Bovine chymotrypsin complexed to bpti |
| 414 | SKEMPI2 | 1REW | AB | C | -12.51 | Structural refinement of the complex of bone morphogenetic protein 2 |
| 415 | SKEMPI2 | 4E6K | AB | G | -7.4 | 2.0 a resolution structure of pseudomonas aeruginosa bacterioferritin |
| 416 | SKEMPI2 | 1DVF | AB | CD | -10.86 | Idiotopic antibody d1.3 fv fragment-antiidiotopic antibody e5.2 fv |
| 417 | SKEMPI2 | 1AK4 | A | D | -6.4 | Human cyclophilin a bound to the amino-terminal domain of hiv-1 capsid |
| 418 | SKEMPI2 | 4O27 | A | B | -10.37 | Crystal structure of mst3-mo25 complex with wif motif |
| 419 | SKEMPI2 | 1XGT | AB | C | -9.43 | Structure for antibody hyhel-63 y33l mutant complexed with hen egg |
| 420 | SKEMPI2 | 4KRL | A | B | -9.08 | Nanobody/vhh domain 7d12 in complex with domain iii of the |
| 421 | SKEMPI2 | 1Z7X | W | X | -18.42 | X-ray structure of human ribonuclease inhibitor complexed with |
| 422 | SKEMPI2 | 4JFF | ABC | DE | -12.57 | Preservation of peptide specificity during tcr-mhc contact dominated |
| 423 | SKEMPI2 | 1MLC | AB | E | -11.39 | Monoclonal antibody fab d44.1 raised against chicken egg- |
| 424 | SKEMPI2 | 1CHO | EFG | I | -15.17 | Crystal and molecular structures of the complex of alpha-*chymotrypsin |
| 425 | SKEMPI2 | 4YFD | A | B | -8.41 | Crystal structure ptp delta ig1-fn2 in complex with il-1racp |
| 426 | SKEMPI2 | 1MI5 | ABC | DE | -6.69 | The crystal structure of lc13 tcr in complex with hlab8-ebv |
| 427 | SKEMPI2 | 1FCC | A | C | -9.03 | Crystal structure of the c2 fragment of streptococcal |

**Table S2.** List of structures from the protein data bank (PDB) used in this work used to validate (external data set) the machine learning methods, and the corresponding experimental binding free energy.

| | Database | PDB ID | Partner1 | Partner2 | $\Delta G_{bind}$ | Description |
|---|---|---|---|---|---|---|
| 1 | BENCHMARK-5.5 | 1XU1 | ABD | T | -11.2 | The crystal structure of april bound to taci |
| 2 | BENCHMARK-5.5 | 1GCQ | B | C | -6.5 | Crystal structure of vav and grb2 sh3 domains |
| 3 | BENCHMARK-5.5 | 1Z0K | A | B | -6.98 | Structure of gtp-bound rab4q67l gtpase in complex with the central rab |
| 4 | BENCHMARK-5.5 | 1E96 | A | B | -7.6 | Structure of the rac/p67phox complex |
| 5 | BENCHMARK-5.5 | 1OPH | A | B | -11.32 | Non-covalent complex between alpha-1-pi-pittsburgh and s195a trypsin |
| 6 | BENCHMARK-5.5 | 3F1P | A | B | -5.35 | Crystal structure of a high affinity heterodimer of hif2 alpha and |
| 7 | BENCHMARK-5.5 | 2SNI | E | I | -15.96 | Structural comparison of two serine proteinase-protein inhibitor |
| 8 | BENCHMARK-5.5 | 1R0R | E | I | -14.37 | 1.1 angstrom resolution structure of the complex between |
| 9 | PDBbind | 1ZC4 | A | D | -10.06 | Crystal structure of the ral-binding domain of exo84 in complex with |
| 10 | PDBbind | 4P3Y | A | B | -9.33 | Crystal structure of acinetobacter baumannii dsba in complex with ef- |
| 11 | PDBbind | 4PZ6 | A | Q | -9.11 | Pce1 guanylyltransferase bound to ser2/ser5 phosphorylated rna pol ii |
| 12 | PDBbind | 4AYI | A | D | -11.87 | Structure of a complex between ccps 6 and 7 of human complement |
| 13 | PDBbind | 5MK0 | A | B | -7.52 | Crystal structure of the his domain protein tyrosine phosphatase (hd- |
| 14 | PDBbind | 5N88 | H | E | -8.18 | Crystal structure of antibody bound to viral protein |
| 15 | PDBbind | 4A49 | A | B | -5.97 | Structure of phosphotyr371-c-cbl-ubch5b complex |
| 16 | PDBbind | 4XXW | B | C | -7.22 | Crystal structure of mouse cadherin-23 ec1-2 and protocadherin-15 ec1- |
| 17 | PDBbind | 2V9T | A | B | -11.03 | Complex between the second lrr domain of slit2 and the |
| 18 | PDBbind | 6F0F | A | B | -9.2 | Crystal structure asf1-ip2_s |
| 19 | PDBbind | 4CMM | A | B | -8.32 | Structure of human cd47 in complex with human signal regulatory |
| 20 | PDBbind | 5F5O | A | B | -9.26 | Crystal structure of marburg virus nucleoprotein core domain bound to |
| 21 | PDBbind | 4NSO | A | B | -12.29 | Crystal structure of the effector-immunity protein complex |
| 22 | PDBbind | 4PAS | A | B | -9.41 | Heterodimeric coiled-coil structure of human gaba(b) receptor |
| 23 | PDBbind | 3MZG | A | B | -3.14 | Crystal structure of a human prolactin receptor antagonist in complex |
| 24 | PDBbind | 3NCC | A | B | -0.92 | A human prolactin receptor antagonist in complex with the mutant |
| 25 | PDBbind | 6F0G | A | C | -9.9 | Crystal structure asf1-ip3 |
| 26 | PDBbind | 6GHO | A | B | -8.32 | Crystal structure of spx in complex with yjbh |
| 27 | PDBbind | 6ERE | B | C | -9.74 | Crystal structure of a computationally designed colicin endonuclease |
| 28 | PDBbind | 4GN4 | B | A | -9 | Obody am2ep06 bound to hen egg-white lysozyme |
| 29 | PDBbind | 1Y3C | E | I | -14.46 | Crystal structure of the complex of subtilisin bpn' with chymotrypsin |
| 30 | PDBbind | 5JDS | B | A | -11.63 | Crystal structure of pd-l1 complexed with a nanobody at 1.7 angstron |
| 31 | PDBbind | 1TO1 | E | I | -12.74 | Crystal structure of the complex of subtilisin bpn' with chymotrypsin |
| 32 | PDBbind | 3UKZ | B | C | -11.76 | Mouse importin alpha: mouse cbp80 cnls complex |
| 33 | PDBbind | 5HU3 | A | B | -8.19 | Drosophila camkii-d136n in complex with a phosphorylated fragment of |
| 34 | PDBbind | 4HEP | A | G | -8.9 | Complex of lactococcal phage tp901-1 with a llama vhh (vhh17) binder |
| 35 | PDBbind | 3BX1 | A | C | -11.39 | Complex between the barley alpha-amylase/subtilisin inhibitor and the |
| 36 | PDBbind | 1TAW | A | B | -14.6 | Bovine trypsin complexed to appi |
| 37 | PDBbind | 5E3E | A | B | -10.08 | Crystal structure of cdia-ct/cdii complex from y. Kristensenii 33638 |
| 38 | PDBbind | 4D0N | A | B | -6.47 | Akap13 (akap-lbc) rhogef domain in complex with rhoa |
| 39 | PDBbind | 4A1S | B | E | -11.32 | Crystallographic structure of the pins:insc complex |
| 40 | PDBbind | 1F3V | A | B | -6.97 | Crystal structure of the complex between the n-terminal domain of |
| 41 | PDBbind | 1PK1 | A | B | -9.91 | Hetero sam domain structure of ph and scm. |
| 42 | PDBbind | 6E3J | A | B | -10.72 | Human bfl-1 in complex with the bfl-1-specific designed peptide |
| 43 | PDBbind | 1G9I | E | I | -9.44 | Crystal structure of beta-trysin complex in cyclohexane |
| 44 | PDBbind | 4C4K | T | O | -7.6 | Crystal structure of the titin m10-obscurin ig domain 1 complex |
| 45 | PDBbind | 3L33 | A | E | -9.37 | Human mesotrypsin complexed with amyloid precursor protein |
| 46 | PDBbind | 2TGP | Z | I | -7.67 | The geometry of the reactive site and of the peptide groups |
| 47 | PDBbind | 4MP0 | A | B | -11 | Structure of a second nuclear pp1 holoenzyme crystal form 2 |
| 48 | PDBbind | 2UYZ | A | B | -9.67 | Non-covalent complex between ubc9 and sumo1 |
| 49 | PDBbind | 3KJ1 | A | B | -11.87 | Mcl-1 in complex with bim bh3 mutant i2da |
| 50 | PDBbind | 5V5H | A | B | -9.28 | Otu protease of crimean congo hemorrhagic fever virus bound to |

| | | | | | | |
|---|---|---|---|---|---|---|
| 51 | PDBbind | 1L0Y | A | B | -7.12 | T cell receptor beta chain complexed with superantigen spea soaked |
| 52 | PDBbind | 1T63 | A | B | -6.58 | Crystal structure of the androgen receptor ligand binding domain with |
| 53 | PDBbind | 4NL9 | A | C | -9.01 | Crystal structure of the human anks3-sam/anks6-sam heterodimer |
| 54 | PDBbind | 4M1L | A | B | -9.2 | Complex of iqcg and ca2+-bound cam |
| 55 | PDBbind | 2IYB | A | E | -7.43 | Structure of complex between the 3rd lim domain of tes and the evh1 |
| 56 | PDBbind | 4LYL | A | B | -10.24 | Crystal structure of uracil-dna glycosylase from cod (gadus morhua) in |
| 57 | PDBbind | 4G01 | A | B | -7.46 | Ara7-gdp-ca2+/vps9a |
| 58 | PDBbind | 5VMO | A | B | -8.26 | Crystal structure of grouper iridovirus giv66:bim complex |
| 59 | PDBbind | 1LP1 | A | B | -7.77 | Protein z in complex with an in vitro selected affibody |
| 60 | PDBbind | 3TZ1 | A | B | -9.35 | Crystal structure of the ca2+-saturated c-terminal domain of akazara |
| 61 | PDBbind | 2IY1 | A | B | -8.33 | Senp1 (mutant) full length sumo1 |
| 62 | PDBbind | 2Z8W | A | D | -11.36 | Structure of an ignar-ama1 complex |
| 63 | PDBbind | 6IUA | A | B | -11.87 | Crystal structure of importin-alpha1 bound to the 53bp1 nuclear |
| 64 | PDBbind | 3U82 | A | B | -10.78 | Binding of herpes simplex virus glycoprotein d to nectin-1 exploits |
| 65 | PDBbind | 2B11 | A | B | -7.23 | Crystal structure of the protein-protein complex between f82w |
| 66 | PDBbind | 5M2O | A | B | -11.38 | R. Flavefaciens' third scab cohesin in complex with a group 1 dockerin |
| 67 | PDBbind | 2NU1 | E | I | -12.77 | Molecular structures of the complexes of sgpb with omtky3 aromatic p1 |
| 68 | PDBbind | 1MXE | A | E | -16.37 | Structure of the complex of calmodulin with the target |
| 69 | PDBbind | 3K1R | A | B | -12.28 | Structure of harmonin npdz1 in complex with the sam-pbm of sans |
| 70 | PDBbind | 1MCV | A | I | -9.56 | Crystal structure analysis of a hybrid squash inhibitor in |
| 71 | PDBbind | 6K06 | A | C | -9.06 | Crystal structure of importin-alpha and phosphomimetic gm130 |
| 72 | PDBbind | 3TKL | A | B | -8.69 | Crystal structure of the gtp-bound rab1a in complex with the coiled- |
| 73 | PDBbind | 1XG2 | A | B | -11.32 | Crystal structure of the complex between pectin |
| 74 | PDBbind | 1Y4A | E | I | -12.17 | Crystal structure of the complex of subtilisin bpn' with chymotrypsin |
| 76 | PDBbind | 3KUD | A | B | -7.87 | Complex of ras-gdp with rafrbd(a85k) |
| 77 | PDBbind | 3C4P | A | B | -11.4 | Crystal structure of the shv-1 beta-lactamase/beta-lactamase inhibitor |
| 78 | PDBbind | 3GMW | A | B | -14.05 | Crystal structure of beta-lactamse inhibitory protein-i (blip-i) in |
| 79 | PDBbind | 5Y4R | A | D | -10.29 | Structure of a methyltransferase complex |
| 80 | PDBbind | 1TA3 | A | B | -10.98 | Crystal structure of xylanase (gh10) in complex with inhibitor (xip) |
| 81 | PDBbind | 6HAR | A | E | -13.94 | Crystal structure of mesotrypsin in complex with appi-m17c/i18f/f34c |
| 82 | PDBbind | 5KY5 | A | B | -8.33 | Mouse pofut1 in complex with egf(+) and gdp |
| 83 | PDBbind | 1TM5 | E | I | -14.69 | Crystal structure of the complex of subtilisin bpn' with chymotrypsin |
| 84 | PDBbind | 1ZV5 | L | A | -10.91 | Crystal structure of the variable domain of the camelid heavy-chain |
| 85 | PDBbind | 5K39 | A | B | -11.32 | The type ii cohesin dockerin complex from clostridium thermocellum |
| 86 | PDBbind | 2P45 | A | B | -9.46 | Complex of a camelid single-domain vhh antibody fragment |
| 88 | PDBbind | 3EJH | A | E | -7.23 | Crystal structure of the fibronectin 8-9fni domain pair in complex |
| 89 | PDBbind | 2XTT | A | B | -14.6 | Bovine trypsin in complex with evolutionary enhanced schistocerca |
| 90 | PDBbind | 6JHW | A | B | -10.57 | Structure of anti-crispr acriic3 and nmecas9 hnh |
| 91 | PDBbind | 6NE2 | B | A | -12.12 | Designed repeat protein in complex with fz7 |
| 92 | PDBbind | 2VOI | A | B | -12.22 | Structure of mouse a1 bound to the bid bh3-domain |
| 93 | PDBbind | 1UUG | A | B | -8.03 | Escherichia coli uracil-dna glycosylase:inhibitor complex |
| 94 | PDBbind | 4U32 | X | A | -8.76 | Human mesotrypsin complexed with hai-2 kunitz domain 1 |
| 95 | PDBbind | 3N06 | A | B | -3.42 | A mutant human prolactin receptor antagonist h27a in complex with the |
| 96 | PDBbind | 1S0W | A | C | -9.24 | 1b lactamse/ b lactamase inhibitor |
| 97 | PDBbind | 4DH2 | A | B | -10.21 | Crystal structure of coh-olpc(cthe_0452)-doc435(cthe_0435) complex: a |
| 98 | PDBbind | 1P6A | A | B | -10.17 | Structural basis for variation in adenovirus affinity for the cellular |
| 99 | PDBbind | 3C4O | A | B | -11.37 | Crystal structure of the shv-1 beta-lactamase/beta-lactamase inhibitor |
| 100 | PDBbind | 5C67 | A | E | -13.71 | Human mesotrypsin in complex with amyloid precursor protein inhibitor |
| 101 | SKEMPI2 | 3SZK | AB | C | -10.06 | Crystal structure of human methaemoglobin complexed with the first |
| 102 | SKEMPI2 | 4HFK | A | BD | -13.05 | Crystal structure of the type vi effector-immunity complex tae4-tai4 |
| 103 | SKEMPI2 | 2J1K | C | T | -12.22 | Cav-2 fibre head in complex with car d1 |
| 104 | SKEMPI2 | 4L3E | ABC | DE | -10.39 | The complex between high affinity tcr dmf5(alpha-d26y;beta-L98W) |
| 105 | SKEMPI2 | 2AK4 | ABC | DE | -6.45 | Crystal structure of sb27 tcr in complex with hla-b*3508- |
| 106 | SKEMPI2 | 1PPF | E | I | -13.16 | X-ray crystal structure of the complex of human leukocyte elastase |

| | | | | | | |
|---|---|---|---|---|---|---|
| 107 | SKEMPI2 | 4MYW | A | B | -10.53 | Structure of hsv-2 gd bound to nectin-1 |

**Table S3.** List of scoring functions and descriptors used to train the super model. All descriptions were taken from the Rosetta software website (https://www.rosettacommons.org/).

| Rosetta Score term (descriptor) | Description |
|---|---|
| dslf_fa13 | Disulfide geometry potential |
| fa_atr | Lennard-Jones attractive between atoms in different residues |
| fa_dun_dev | Internal energy of sidechain rotamers as derived from Dunbrack's statistics involving the deviation of $\chi$ angles from rotamericity |
| fa_dun_rot | Internal energy of sidechain rotamers as derived from Dunbrack's statistics involving the frequency of rotamers |
| fa_dun_semi | Counterpart of fa_dun_rot for semi-rotameric amino acids |
| fa_elec | Coulombic electrostatic potential with a distance-dependent dielectric |
| fa_intra_atr_xover4 | Intra-residue LJ attraction counted for the atom-pairs beyond torsion-relationship. |
| fa_intra_elec | Intra-residue Coulombic interaction counted for the atom-pairs beyond torsion-relationship |
| fa_intra_rep_xover4 | Intra-residue LJ repulsion counted for the atom-pairs beyond torsion-relationship |
| fa_intra_sol_xover4 | Intra-residue LK solvation counted for the atom-pairs beyond torsion-relationship |
| fa_rep | Lennard-Jones repulsive between atoms in different residues |
| fa_sol | Lazaridis-Karplus solvation energy |
| hbond_bb_sc | Sidechain-backbone hydrogen bond energy |
| hbond_lr_bb | Backbone-backbone hbonds distant in primary sequence |
| hbond_sc | Sidechain-sidechain hydrogen bond energy |
| hbond_sr_bb | Backbone-backbone hbonds close in primary sequence |
| hxl_tors | Sidechain hydroxyl group torsion preference for Ser/Thr/Tyr supersedes yhh_planarity (that covers L- and D-Tyr only) |
| lk_ball | Anisotropic contribution to the solvation. |
| lk_ball_bridge | Bonus to solvation coming from bridging waters measured by overlap of the 'balls' from two interacting polar atoms. Supports arbitrary residue types. |
| lk_ball_bridge_uncpl | Same as 'lk_ball_bridge' but the value is uncoupled with dGfree (i.e., constant bonus whereas lk_ball_bridge is proportional to dGfree values). Supports arbitrary residue types. |
| lk_ball_iso | Same as fa_sol; see below. Supports arbitrary residue types. |
| omega | Omega dihedral in the backbone. A Harmonic constraint on planarity with standard deviation of ~6 deg. Supports alpha-amino acids beta-amino acids and oligoureas. In the case of oligoureas both amide bonds (called 'mu' and 'omega' in Rosetta) are constarined to planarity. |
| p_aa_pp | Probability of amino acid at ฮฆ/ฮจ. |
| pro_close | Proline ring closure energy and energy of psi angle of preceding residue. Supports D- or L-proline plus D- or L-oligourea-proline |
| rama_prepro | Backbone torsion preference term that takes into account of whether preceding amino acid is proline or not. |
| ref | Reference energy for each amino acid. Balances internal energy of amino acid terms. Plays role in design. |
| total_score | Sum of the individual residue scores in Rosetta. |
| shape complementarity (sc) | Calculates the Lawrence & Coleman shape complementarity. |
| ddg | Computes the binding energy for the complex |
| interactionEnergyMetric | A metric for measuring the short- and long-range interaction energy between residues using two sets of residue selectors. |
| complex_normalized | Average energy of a residue in the entire complex |
| dG_cross | Binding energy of the interface calculated with cross-interface energy terms, rather than by separating the interface. Inaccurate sometimes because of environmental dependencies in some energy terms, including hbond energy and solvation |
| dG_cross/dSASAx100 | dG_cross divided by dSASA, multipled by 100 |
| dG_separated | The change in Rosetta energy when the interface forming chains are separated, versus when they are complexed: the binding energy. Calculated by actually separating (and optionally repacking) the chains |
| dG_separated/dSASAx100 | Separated binding energy per unit interface area 100 to make units fit in score file. Scaling by dSASA controls for large interfaces having more energy. The factor of 100 is to allow standard 2.45 notation instead of something like 2.45E-2 |

| | |
|---|---|
| dSASA_hphobic | The nonpolar part of FACTS solvation free energy, which was parametrized to approximate atomic solvent accessible surface area, in Å$^2$ |
| dSASA_int | The solvent accessible area burried at the interface, in Å$^2$ |
| dSASA_polar | The change in Rosetta energy when the interface forming chains are separated, versus when they are complexed: the binding energy. Calculated by separating (and optionally repacking) the chains |
| delta_unsatHbonds | The number of buried unsatisfied hydrogen bonds at the interface |
| hbond_E_fraction | Amount of interface energy (dG_separated) accounted for by cross interface H-bonds |
| hbonds_int | Total cross-interface hydrogen bonds found. |
| nres_all | Total number of residues in the entire complex. |
| nres_int | Number of residues at the interface. |
| packstat | Rosetta's packing statistic score for the interface (0=bad, 1=perfect). |
| per_residue_energy_int | Average energy of each residue at the interface. |
| sc_value | Shape complementarity. |
| side1_normalized | Average per-residue energy on one side of the interface. |
| side1_score | Energy of one side of the interface. |
| side2_normalized | Average per-residue energy on the other side of the interface. |
| side2_score | Energy of the other side of the interface. |

| **Models** | **Random Split of Target Function*** | | **Gaussian Split of Target Function** | |
|---|---|---|---|---|
| | RMSE (kcal/mol) | R | RMSE (kcal/mol) | R |
| **PRODIGY** | 2.69 | 0.35 | 3.11 | 0.16 |
| **Rosetta** | 53.91 | 0.11 | 56.23 | 0.07 |
| **LR** | 2.49 | 0.52 | 2.17 | 0.62 |
| **EN** | 2.45 | 0.56 | 2.20 | 0.60 |
| **SV** | 2.77 | 0.34 | 2.72 | 0.16 |
| **DT** | 2.91 | 0.43 | 2.94 | 0.44 |
| **KN** | 2.46 | 0.54 | 2.41 | 0.52 |
| **AD** | 2.42 | 0.60 | 2.26 | 0.56 |
| **BG** | 2.23 | 0.68 | 2.17 | 0.63 |
| **RF** | 2.20 | 0.69 | 2.12 | 0.65 |
| **ET** | 2.11 | 0.72 | 1.97 | 0.70 |
| **XB** | 2.21 | 0.65 | 2.32 | 0.59 |
| **SL** | 2.18 | 0.68 | 1.98 | 0.70 |

* Data obtained

**Theoretical description of SHAP (SHapley Additive exPlanations)**

SHAP (SHapley Additive exPlanations) is a framework for explaining the output of machine learning models by assigning contributions to each feature of the input data. Given a prediction model $f$ and an instance $x$ with $N$ features, SHAP values ($\phi$) aim to decompose the model's prediction ($f(x)$) into contributions from individual features. Formally, SHAP values satisfy the following properties:

1. Local accuracy: The sum of SHAP values for a specific instance equals the model's prediction for that instance:

$$\sum_{i=1}^{N} \phi_i(x) = f(x)$$

2. Fairness: If two features are identical in all respects except for the feature of interest, their SHAP values should be the same.
3. Additivity: SHAP values can be added together across features to obtain the prediction difference between two instances:

$$f(x') - f(x) = \sum_{i=1}^{N} \phi_i(x') - \phi_i(x)$$

4. Consistency: a less important feature is added to a model, its SHAP value should not increase for any instance:

$$\phi_i(z) \leq \phi_i(x) \; if \; z_i \leq x_i \; \forall \; i$$

The computation of SHAP values involves creating a reference or baseline instance ($\phi_0$) and considering all possible feature permutations. The idea is to calculate the average contribution of each feature across all possible orders of adding features. Mathematically, the SHAP value for feature $i$ is defined as:

$$\phi_i(x) = \frac{1}{N!} \sum\nolimits_{\mathcal{P} \in S} [f_{\mathcal{P}(i)}(x_{\mathcal{P}}) - f(x)$$

Where $N$ is the number of features, $S$ is the set of all permutations, is $f_{\mathcal{P}(i)}(x_{\mathcal{P}})$ the model prediction when feature $i$ is added to the permutation $\mathcal{P}$, and $x_{\mathcal{P}}$ is the instance with features permuted according to $\mathcal{P}$.
